# Feasibility and Reliability Assessment of Video-Based Motion Analysis and Surface Electromyography in Children with Fragile X during Gait

**DOI:** 10.3390/s21144746

**Published:** 2021-07-12

**Authors:** Zimi Sawacha, Fabiola Spolaor, Weronika Joanna Piątkowska, Federica Cibin, Alfredo Ciniglio, Annamaria Guiotto, Marco Ricca, Roberta Polli, Alessandra Murgia

**Affiliations:** 1 Department of Information Engineering, University of Padova, 35131 Padova, Italy; zimi.sawacha@dei.unipd.it (Z.S.); fabiola.spolaor@unipd.it (F.S.); weronikajoanna.piatkowska@studenti.unipd.it (W.J.P.); fede.cibin90@gmail.com (F.C.); ciniglioal@dei.unipd.it (A.C.); guiotto@dei.unipd.it (A.G.); 2 Department of Medicine, DIMED, University of Padova, 35121 Padova, Italy; 3 Department of Women’s and Children’s Health, University of Padova, 35128 Padova, Italy; ricca.m@gmail.com (M.R.); roberta.polli@unipd.it (R.P.); 4 Istituto di Ricerca Pediatrica CDS, 35127 Padova, Italy

**Keywords:** gait analysis, fragile X syndrome, surface electromyography, kinematics

## Abstract

Fragile X Syndrome (FXS), the leading form of inherited intellectual disability and autism, is characterized by specific musculoskeletal conditions. We hypothesized that gait analysis in FXS could be relevant for the evaluation of motor control of gait, and help the understanding of a possible correlation between functional and intellectual abilities. Typical deficits in executive control and hyperactivity have hampered the use of standard gait analysis. The aim of our study was to quantitatively assess musculoskeletal alterations in FXS children in standard ambulatory conditions, in a friendly environment. Ten FXS children and sixteen controls, with typical neurodevelopment, were evaluated through four synchronized video cameras and surface electromyography; lower limb joints rotations, spatiotemporal parameters, duration of muscle contraction, activation timing and envelope peaks were determined. Reliability and repeatability of the video based kinematics analysis was assessed with respect to stereophotogrammetry. The Kruskal–Wallis Test (*p* < 0.05) or SPM1D were used to compare different groups of subjects. Results show a consistently altered gait pattern associated with abnormal muscle activity in FXS subjects: reduced knee and excessive hip and ankle flexion, and altered duration and activity onset on all the recorded muscles (Rectus/Biceps Femoris, Tibialis Anterior, Gastrocnemius Lateralis). Results of this study could help with planning personalized rehabilitations.

## 1. Introduction

Fragile X Syndrome (FXS) is the leading cause of inherited intellectual disability (ID) and autism spectrum disorder (frequency estimated at 1/4000–1/7000) [1]. This condition is one of several distinct phenotypes associated with pathologic expansions of the polymorphic region of CGG repeats of the *FMR1* gene. These expansions fall into two main mutational categories generating opposite pathogenic mechanisms. In the “premutation” (55–200 CGGs), a great increase in gene expression is key to the cell toxicity responsible for the so called FX-associated phenotypes: Fragile X-associated Primary Ovarian Insufficiency (FXPOI), Fragile X-associated Tremor Ataxia Syndrome (FXTAS), and Fragile X-associated Neuropsychiatric Disorder (FXAND). In the “full mutation”, an expansion of the CGG repeated region beyond 200 elements triggers the methylation of the gene promoter and of the entire region of repeats, which results in transcriptional silencing and loss of protein expression [2]. It is the loss of FMR1 gene expression, the opposite mechanism with respect to the associated conditions, which is responsible for the Fragile X Syndrome phenotype. Somatic mosaicism for pre and full mutation, a relatively common occurrence, can be a strong phenotype modulator of the FXS clinical manifestations [3]. FXS affects both sexes, although in females the phenotype is usually milder as a consequence of the physiologic X inactivation phenomenon [2]. 

In FXS children, characteristic musculoskeletal manifestations, which include hypotonia, joint laxity and flexible flat feet [4], may lead to non-physiological gait patterns. In other neurodevelopmental conditions (i.e., Down, Williams, Ehlers–Danlos or Prader–Willi syndromes) with partially similar clinical features, gait analysis has documented significant motor alterations. For instance, in subjects with Down, Prader–Willi and Williams syndromes, reduced walking speed with short steps, increased knee and/or hip flexion and reduced ankle joint excursion during the rolling of the ankle and forefoot were observed, together with increased intra-subject variability of the walking pattern. These syndromes are characterized by both physical and cognitive impairments, and therefore both aspects are considered responsible for their gait alterations. In subjects with Ehlers–Danlos syndrome, a lower dorsiflexion both in the stance and the swing phases was found to be associated with weakness of the Tibialis Anterior and Gastrocnemius Lateralis, joint stiffness, joint laxity and hypotonia [5,6,7,8]. All these neurodevelopmental conditions, characterized by flat foot associated with joint laxity and hypotonia, lead to an increase in the activity of the Tibialis Anterior and a decrease in the activity of the Peroneus Longus [9]. Differences in muscle activity in people with flat-foot may reflect neuromuscular compensation to reduce the overload at the medial longitudinal arch [9]. 

We hypothesized that, despite the obvious difficulties in performing gait analysis in subjects with neurodevelopmental conditions with ID, we could detect specific alterations in the motor control of gait in a population of FXS children. The assessment of these characteristics could help with planning personalized rehabilitation. 

In children with FXS, severe deficits in executive control and visuospatial abilities, a high degree of anxiety, hyperactivity and other behavioural problems, have hampered the application of state-of-the-art gait analysis by means of force plates, stereophotogrammetry and surface electromyography (sEMG) [10,11]. For these reasons, a video-based gait analysis coupled with sEMG has been adopted. Similarly, a markerless motion analysis approach was adopted to monitor the frequency of movements in a sample of FXS subjects in order to characterize the phenomenon of hyperkinesis; however, no gait analysis data were collected [11]. 

In the present study, a video-based methodology, similar to the one adopted to study the gait of a cohort of Parkinson’s Disease subjects in underwater conditions [12], was used. The adopted automatic feature tracking software was originally developed to work in underwater conditions in order to assess the kinematics of swimmers, and its reliability was tested against a commercial automatic feature tracking software [13]. In the present study, a modified version of the automatic tracking algorithm is presented in order to track features applied a posteriori to the video sequences; hence 2D joint lower limb kinematics together with the spatiotemporal parameters are assessed in a cohort of children with and without FXS during gait. 

Our study had two main aims: 1. to verify the feasibility of gait analysis in FXS individuals with the use of video-based motion analysis and surface electromyography (sEMG), in a routine clinical follow up within standard ambulatory conditions; and 2. to quantitatively assess gait alterations in FXS with respect to a group of healthy subjects. 

As a secondary aim, both the reliability and repeatability of the proposed video-based methodology were assessed with respect to state-of-the-art gait analysis [14] performed by means of a stereophotogrammetric system on a group of children with typical neurodevelopment. 

## 2. Materials and Methods

### 2.1. Population 

The study was conducted according to the local Ethics Committee recommendations (Università Azienda Ospedaliera di Padova, trial *n*° 46039, date of registration 29 July 2019). Regular informed consent was obtained for each participant for the scientific use of the data and publication. Power analyses using ankle, knee, and hip kinematic data in [15] indicated that three to nine participants per group would be needed for comparisons between subjects with autism spectrum disorder and controls [15] and in the same population from four to eight participants would be needed to find speed main effects [16,17]; the following equations were applied to calculate the necessary number of subjects for our study as in [18]:

(1) $$n=\frac{2}{d^{2}}\times c_{p,power},\;$$

where *n* is the number of subjects required in the group, *d* is the standardized difference and *c_p_*_,*power*_ is a constant defined by the values chosen for the *p* value and power. The formula was applied to the gait velocity of a preliminary dataset [19] from our group (see Supplementary Materials) composed of four FXS children and ten controls:

(2) $$n=\frac{2}{0.78^{2}}\times c_{0.05,80\%}.\;$$

Due to the fact that the preliminary study had an unequal sample size, the calculated number of subjects was adjusted for a non-equal sample size:

(3) $$N^{\prime }=\frac{N{\left(1+k\right)}^{2}}{4k},\;$$

where *N*′ is the revised total sample size, *N* is total sample size calculated using Equation (1) and *k* is the actual ratio of the two groups.

Equation (3) showed that a total of 9 subjects was sufficient for our analysis; we therefore enrolled 12 FXS children and 19 controls for the present study. 

Twelve participants with FXS were evaluated (see Table 1) in a routine clinical setting at the Department of Women’s and Children’s Health, University of Padova; 7 carried a classical full mutation of the FMR1 gene, and 5 carried a full mutation of the FMR1 gene in a state of size (3) or methylation (2) mosaicism. All of these children presented ID as well as ligamentous laxity and flat foot. 

FXS subjects were enrolled for the study according to the following inclusion/exclusion criteria:

1. Molecularly documented full mutation of the FMR1 gene with expansions of more than 200 CGG repeats and methylation of the promoter and repeated sequence; possible size and/or methylation mosaicism;
2. Ability to walk independently;
3. Absence of documented orthopaedic comorbidities affecting the lower limbs within 12 months from the beginning of the study;
4. Absence of documented neurological disorders.

Nineteen controls, matched for Body Mass Index and age (see Table 1) and with typical neurodevelopment, were evaluated at the Bioengineering of Movement Laboratory of the Department of Information Engineering, University of Padova. Among the controls, we found a documented flat foot in 6 children and ligamentous laxity in 3. 

Controls were enrolled according to the following inclusion/exclusion criteria:

1. Ability to walk independently;
2. Absence of documented lower limbs injures within 12 months from the beginning of the study;
3. Absence of documented neurological disorders.

Within the control group, three different subgroups were identified (based on documented professional diagnosis by orthopaedic doctors) in order to distinguish the possible influence of flat foot and ligamentous laxity in the gait pattern as follows: control subjects without any foot deformities or the presence of ligamentous laxity (CS), controls with flat foot (CSF) and controls with ligamentous laxity (CSL). 

### 2.2. Molecular Analysis 

Molecular analysis was performed as previously reported [20,21,22]. Genomic DNA (gDNA) was extracted from peripheral blood leukocytes (PBL) and saliva on an automated Maxwell^®^ 16 Blood DNA Purification System (Promega, Milan, Italy) and quantified by spectrophotometer with NanoDrop^TM^ (ThermoFisher Scientific, Waltham, MA, USA). To identify the full range of FMR1 CGG repeat expansions, genomic DNA (40–60 ng) was amplified with an Amplidex FMR1 PCR kit (Asuragen, Austin, TX, USA) as previously described [23] and according to the manufacturer’s recommended protocol. All amplicons were analysed by capillary electrophoresis (CE) on a 3130*xl* Genetic Analyzer (Applied Biosystems, ThermoFisher Scientific, Waltham, MA, USA). Fragment length was derived from the size of the PCR products calibrated to a ROX 1000 Size Ladder (Asuragen, Austin, TX, USA) with the use of the GeneMapper^®^ Software v 4.0 (ThermoFisher Scientific, Waltham, MA, USA). Methylation analysis was carried out with use of the AmplideX FMR1 mPCR kit (Asuragen, Austin, TX, USA), as described [24]. Methylation percentage was calculated as a ratio of peak heights between digested (HEX) and undigested samples (FAM), normalized to the CGG control amplicon peak height with the GeneMapper^®^ Software v 4.0. Alleles are reported as unmethylated (<20%), partially methylated (20–80%) and fully methylated (>80%). 

### 2.3. Instrumental Assessment

**Figure 1:**
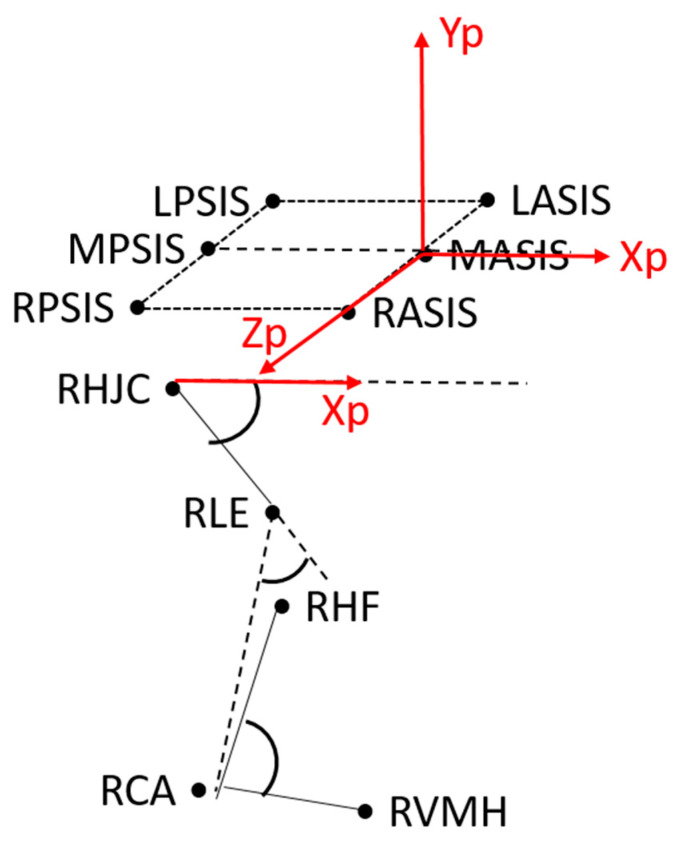
Lower limb embedded frames and sagittal plane angles definition. The pelvis reference system is represented in red: the origin is the midpoint between LASIS and RASIS (MASIS), the z axis is the normalized vector oriented as the line passing through the LASIS and RASIS with its positive direction from left to right; the x axis lies in the plane defined by the RASIS, LASIS and the midpoint between the RPSIS and LPSIS (MPSIS) with its positive direction forwards; the y axis is orthogonal to the xz plane and its positive direction is proximal. The hip flexion angle is defined as the angle between pelvis and femur (femur is the line connecting the hip joint centre (HJC) and the lateral epicondyle (LE)); the knee flexion angle is defined as the angle between femur and shank (shank is the line connecting the LE and the calcaneus (CA)); the ankle flexion angle is defined as the angle between shank and foot (foot is the line connecting the CA and the fifth metatarsal head (VMH)). The following acronyms were used: right and left anterior superior iliac spines (RASIS, LASIS); right and left posterior superior iliac spines (RPSIS, LPSIS); right and left lateral epicondyles (RLE, LLE); right and left heads of fibula (RHF, LHF); right and left calcanei (RCA, LCA); right and left fifth metatarsal heads (RVMH, LVMH); right and left HJC (RHJC, LHJC).

Kinematics and sEMG data were simultaneously acquired through four synchronized cameras (GoPro Hero3 and GoPro Hero7, 1080 × 1920 pixel resolution, 30 fps) and an sEMG system (FreeEmg, BTS, 1000 Hz) that collected the activity of the Tibialis Anterior (TA), Gastrocnemius Lateralis (GL), Rectus Femoris (RF) and Biceps Femoris (BF) bilaterally. Concerning the video-based kinematics analysis, the data on the CS subjects were acquired in four different conditions for validation purposes:

1. Set up 1: a stereophotogrammetric system (6 cameras BTS 60 Hz) was used and reflective markers were applied on anatomical landmarks according to [14,25] (in the following paragraph this will be referred to as the “gold standard” (GS));
2. Set up 2: a video-based system [26] was adopted and reflective markers were applied on anatomical landmarks according to [14,25] (in the following paragraph this will be referred to as the “Marker”);
3. Set up 3: a video-based system was adopted and markers made with double coloured tape were applied on anatomical landmarks according to a simplified version of [27] (in the following paragraph this will be referred to as “Tape”, see Figure 1).
4. Set up 4: a video-based system was adopted without applying any marker (“No Tape”).

Meanwhile, the data on the FXS subjects were acquired without applying any markers onto their skin (set up 4). The calibration of both the cameras’ intrinsic and extrinsic parameters was achieved from the acquisition of a checkerboard pattern (square: 4 mm × 4 mm, pattern: 75 cm × 54.5 cm) [28]. Each subject performed several gait trials and at least six trials; three right and three left gait cycles were processed per subject. A total sample of 687 trials was collected, a subset of which (540 trials) was analysed based on the coefficient of multiple correlation (CMC) value (CMC > 0.75). A total of 147 trials were discarded.

### 2.4. Data Processing

#### 2.4.1. Analysis of Video Sequences

Once acquired, the video sequences were processed in order to extract the three dimensional anatomical landmark coordinates through the software “Track on Field” (BBSoF s.r.l.), which implements the optical flow popular Kanade–Lucas–Tomasi tracking algorithm [29] in the version proposed by Sawacha et al., 2014 [30]. The algorithm explicitly optimizes the tracking performance by classifying a feature as appropriate if it can be tracked successfully. This algorithm was validated in underwater conditions with respect to a commercial video tracking software [13]. In the present contribution, the algorithm was modified in order to enable the tracking of video sequences without the presence of markers in the scene as follows: the user uses the mouse to identify any anatomical landmarks according to the chosen marker set (see Figure 1) and then a marker is added by the software automatically. Afterwards, the marker will be tracked automatically frame by frame, utilizing the information from the previous frame. The key point is to minimize the sum of the squared differences between the local coordinates in the subsequent frames over a suitable neighbourhood. The best match in the next frame can then be found [31]. The tracking process is then iterated by updating the positions of the point.

#### 2.4.2. Kinematics Parameters Extraction

Data were processed (Matlab R.19) and sagittal plane kinematics, as well as spatiotemporal parameters, were extracted according to a simplified version of [14,25], which considers the following anatomical landmarks for retrieving the joint embedded reference system (see Figure 1): right and left anterior (R/LASIS) and posterior superior iliac spines (R/LPSIS); right and left lateral epicondyles (R/LLE); right and left heads of fibula (R/LHF); right and left calcanei (R/LCA); and right and left fifth metatarsal heads (R/LVMH). The hip joint centre (RHJC, LHJC) was calculated as in [32].

Regarding the 2D joint rotation angles (defined in Figure 1), each subject’s variables were represented by the mean from three representative walking trials for the right and left sides. The CMC [33] was used to aid the selection of which subject representative walking trial could be included in the computation of the mean; thus, the coefficient was calculated for each subject’s kinematic parameter. Walking trials, the kinematics variables of which were found to have a CMC of less than 0.75 (75%), were excluded from the statistical analysis [25]. Afterwards, normative bands were created with the data of the CS group as mean and standard deviation. 

#### 2.4.3. Reliability and Repeatability of the Video-Based Gait Analysis Protocol in Children

In order to test both the reliability and the repeatability of the proposed methodology, 10 subjects were selected from the CS group without any orthopaedic alterations, such as flat foot and ligamentous laxity, and their gait was acquired simultaneously with the stereophotogrammetric system (BTS) and the video-based system. Each subject performed several gait trials using four different set-ups (see Section 2.3). 

In the “Gold Standard” step up, anatomical landmarks’ trajectories were reconstructed through a stereophotogrammetric system and joint embedded frames were defined according to [14,25]. In the “Marker” set-up, anatomical landmarks trajectories were reconstructed through an automatic tracking of feature software, Track On Field (BBSoF S.r.l), based on the algorithm developed in [13,34]. In this case, joint rotations were defined according to [14,25]. In the two final set ups (“Tape” and “No Tape”), a simplified version of [27] was applied and joint embedded frames and joint rotations were defined as in Figure 1. 

Three different comparisons were carried out based on the 4 setups, in order to assess the different sources of variability as follows:

- The comparison between set up 1 and 2 allows the assessment of the reliability of the automatic feature tracking software in reconstructing the anatomical landmarks’ positions during gait with respect to a stereophotogrammetric gold standard;
- The comparison between set up 3 and 1 allows the assessment of the role of one side of the type of marker (double coloured double sided tape in set up 3) in reconstructing the anatomical landmarks’ trajectories; on the other one of a reduced marker set on the definition of the joint embedded frames and, consequently, on the joint angles;
- The comparison between setup 4 and 1 allows the assessment of the role of visual identification of the anatomical landmarks, in the absence of markers, on the reconstruction of the anatomical landmarks’ trajectories and, consequently, on the joint angles.

Comparisons were made between the joint angles and not between the anatomical landmarks’ trajectories in order to assess the reliability of the measures, which was the objective of the gait analysis. The estimation of the root mean square distance (RMSD), computed over the 100 samples of the gait cycle as in [35] (see Table 2), was adopted. RMSD was also expressed as a percentage of the Gold Standard measure and was compared with the state-of-the-art [34,35]. For the inter-trial variability assessment, the CMC [33] was calculated for each biomechanical variable. 

Based on previous publications [33,36], the values of CMC were interpreted as follows:

- 0.65–0.75: moderate
- 0.75–0.85: good
- 0.85–0.95: very good
- 0.95–1: excellent

**Figure 2:**
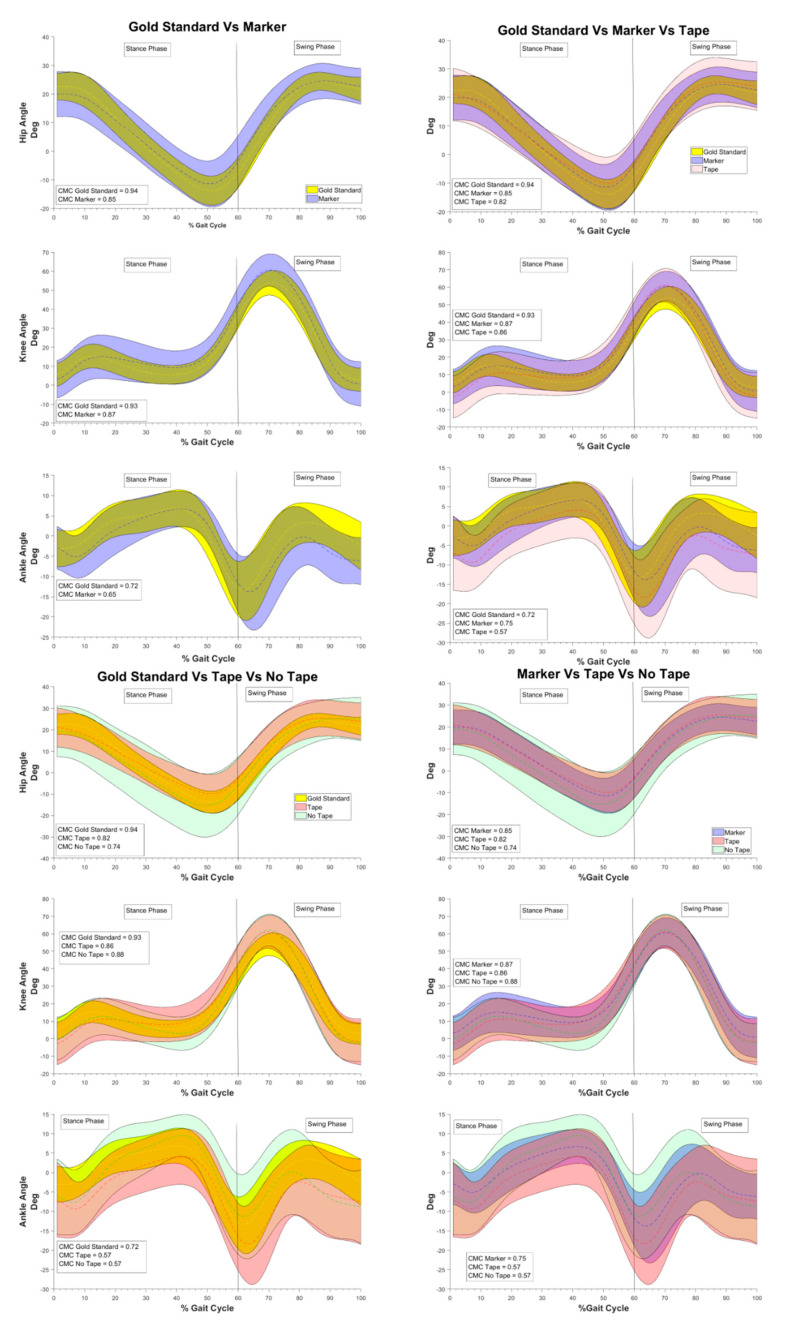
The hip, knee and ankle sagittal plane angles (mean ± SD), for each comparison between the set ups, are represented. The CMC, for each set up, is reported. The set ups are represented as follows: in yellow the “Gold Standard”, in blue “Marker”, in red “Tape” and in green “No Tape”.

To verify the inter-operator variability, two operators performed the video-tracking, and the Standard Error (“SE”) was calculated across measures as in [37] (see Figure 2).

#### 2.4.4. sEMG Data Processing

In terms of sEMG analysis, signal envelope [38], duration initiation and cessation of muscle activity [39] were computed for each gait cycle.

Signals were band pass filtered with a 5th order Butterworth filter and were full wave rectified. To compute the envelope, 10 Hz for the high pass and 450 Hz for the low pass filter were applied. The envelope was obtained by low-pass filtering the signals with a 4th order Butterworth filter and a cut off frequency of 5 Hz [40]. In order to allow comparison across subjects, and by considering the impossibility of acquiring a maximum voluntary contraction on these children within the hospital facility, the peak of each muscle’s sEMG activity was normalized on the mean value within the gait cycle [38]. The occurrence of the envelope peak was extracted with respect to the gait cycle [40].

The durations and intervals of muscle activations and deactivations were calculated [39]. The cut-off frequencies varied from 5 to 15 Hz for high pass filters, and between 450 and 495 Hz for low pass filters. sEMG signals for each muscle were additionally filtered using a filter removing the heartbeat, and a notch filter for a 50 HzA double-threshold statistical detector was applied for signal processing [39] based on the selection of the first threshold ζ and by observing the chosen number of successive samples (m). The signal was detected only if at least the specified number of samples (r0)—which is the second threshold—in the observed interval was above the first threshold. The value of ζ was based on the level or the estimation of the background noise. All three parameters, ζ, r0, and m, were selected to minimize the false-alarm probability value and maximise the detection probability based on the signal-to-noise ratio (SNR) value of each signal. Background noise was estimated for each signal based on the interval of the subject’s inactivity. Only activation intervals longer than 30 ms were accepted as valid muscular contractions [39]. The frequency of activation was defined according to the number of subjects in which a muscle activity at each percentage of the gait cycle was detected [41]. Finally, the data of CS, CSL and CSF were compared with the data of FXS Full Mutation and FXS Mosaics.

#### 2.4.5. Variables Extracted

In terms of spatio-temporal parameters, the following variables were analysed:

- stance time in percentage of the gait cycle;
- stride length (m);
- gait cycle duration (s);
- gait velocity (m/s);
- swing time in percentage of the gait cycle;
- gait cadence (step/min).

In terms of sEMG parameters, the following variables were analysed:

- peak of the envelope;
- peak of the envelope occurrence within the gait cycle;
- envelope profiles;
- duration of muscle activation;
- onset and offset of muscle activation.
- In terms of kinematic parameters, the following variables were analysed:
- 2D joint rotation angles.

### 2.5. Statistical Analysis

Due to the small number of subjects per group, the Kruskal–Wallis non-parametric test (two-tailed α = 0.05), with post-hoc Wilcoxon rank sum test with Bonferroni correction, was used to compare spatiotemporal parameters, peak of the envelope, and its occurrence within the gait cycle among CS, CSF, CSL, FXS Full Mutation and FXS Mosaics groups (SPSS v24, IBM Statistical Toolbox).

Since the sEMG envelope profiles were time series, multiple comparisons tests on pairs of samples, medians were conducted by means of the Kruskal–Wallis tests (two-tailed α = 0.05) with post-hoc Wilcoxon rank sum tests with Bonferroni correction in MATLAB^®^ (v. R2019a). The following groups were compared: CS vs. FXS Full Mutation vs. FXS Mosaics; CSF vs. FXS Full Mutation vs. FXS Mosaics; and CSL vs. FXS Full Mutation vs. FXS Mosaics.

Joint kinematics profiles were analysed using non-parametric 1D statistical parametric mapping (SPM1D) [42], comparing the above mentioned groups. SPM1D’s non-parametric procedures were calculated for each time node and were expressed as SPM1D{t} trajectories. A critical threshold was then defined that only 5% (α = 0.05) of identically smooth random curves were expected to exceed. Parts of the gait cycle where the SPM1D{t} trajectory crossed this threshold were identified as clusters with a significant outcome, for which cluster-specific *p*-values were calculated based on the Random Field Theory [43]. Bonferroni correction for multiple testing brought α to 0.017. All SPM1D analyses were performed using the spm1d open source code (vM.0.4.5, http://www.spm1d.org (accessed on 1 May 2021)) in MATLAB^®^. 

## 3. Results

Characteristic gait alterations were observed in FXS, in both fully mutated and mosaic individuals. In the following sections, the results of the study are reported according to the specific comparison. 

### 3.1. Reliability and Repeatability of Video-Based Motion Analysis in CS

In terms of reliability, the comparison with the Gold Standard (i.e., stereophotogrammetric system) was reported in Figure 2 in terms of CMC, and in Table 2 in terms of mean RMSD over the gait cycle and RMSD% of the Gold Standard value [34,35].

### 3.2. Kinematics Analysis 

**Figure 3:**
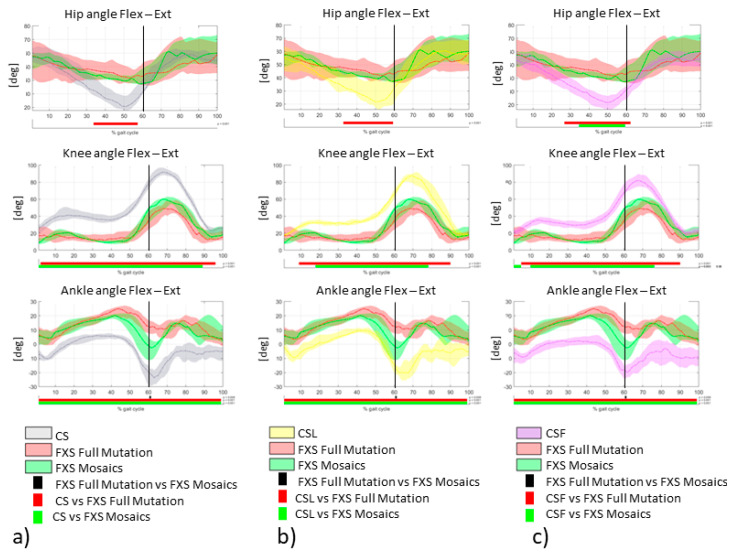
Hip, knee and ankle sagittal plane angles between CS vs. FXS Full Mutation vs. FXS Mosaics (**a**), CSL vs. FXS Full Mutation vs. FXS Mosaics (**b**), CSF vs. FXS Full Mutation vs. FXS Mosaics (**c**); median, 25th and 75th percentile are represented; horizontal bars represent clusters with significant differences (SPM *t*-test).

For methodological purposes, the median and quartile were used in the following graphs since we have conducted non-parametric tests. Individuals with FXS with Full Mutation and Mosaics displayed a pattern of excessively flexed hip (during midstance and push-off) and ankle (over the whole gait cycle), with a reduced knee flexion (over the whole gait cycle). However, the hip kinematics in the FXS Mosaics group showed a pattern closer to CS, independently from the presence of flat foot or ligamentous laxity (Figure 3, Supplementary Materials). Interestingly, a statistically significant difference was only detected between FXS Full Mutation and FXS Mosaics groups during the push off phase of gait at the ankle joint.

**Figure 4:**
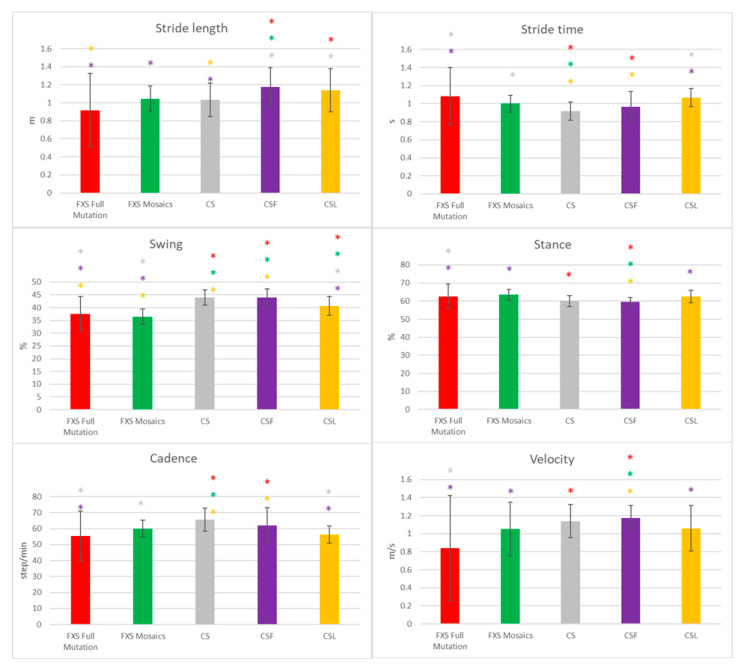
Spatiotemporal parameters of gait in FXS Full Mutation (red), FXS Mosaics (green), CS (grey), CSF (purple), CSL (yellow). Median and interquartile range (IQR) with asterisk (*) when statistically significant differences are observed (*p*-value of the Wilcoxon rank-sum test < 0.05): red with respect to FXS Full Mutation, green with respect to FXS Mosaics, grey with respect to CS, purple with respect to CSF and yellow with respect to CSL. Colours refer to the online version of the figure.

Statistically significant differences were observed in the spatiotemporal gait parameters among the tested groups as follows (Figure 4 and Supplementary Materials):

- Comparison between FXS Full Mutation and FXS Mosaics groups with respect to CS, reduced velocity (in FXS Full Mutation vs. CS), swing duration, and cadence accompanied by increased stride time and stance duration (in FXS Full Mutation vs. CS);
- Comparison between FXS Full mutation and FXS Mosaics groups with respect to CSL reduced swing duration and reduced stride length (in FXS Full Mutation vs. CSL);
- Comparison between FXS Full Mutation and FXS Mosaics groups with respect to CSF, reduced stride length, swing duration and velocity accompanied by increased stance duration; an increased stride time and reduced cadence (in FXS Full Mutation vs. CSF)

### 3.3. sEMG

In FXS individuals with full mutations of the Rectus Femoris and Tibialis Anterior (i.e., muscles of the anterior aspect of lower limbs) were continuously activated and deactivated throughout the gait cycle. In the FXS Mosaics group, the same type of activity was observed on the Right Biceps Femoris and on the Gastrocnemius Lateralis (i.e., muscles of the posterior aspect of lower limbs) bilaterally; while in the FXS Full Mutation group, the same type of activity was observed on the Left Biceps Femoris. Similar alterations were not detected in either CSL or CSF with typical neurodevelopment. The FXS Mosaics group, differently from the FXS Full Mutation group, displayed a muscle activity closer to CS. 

**Figure 5:**
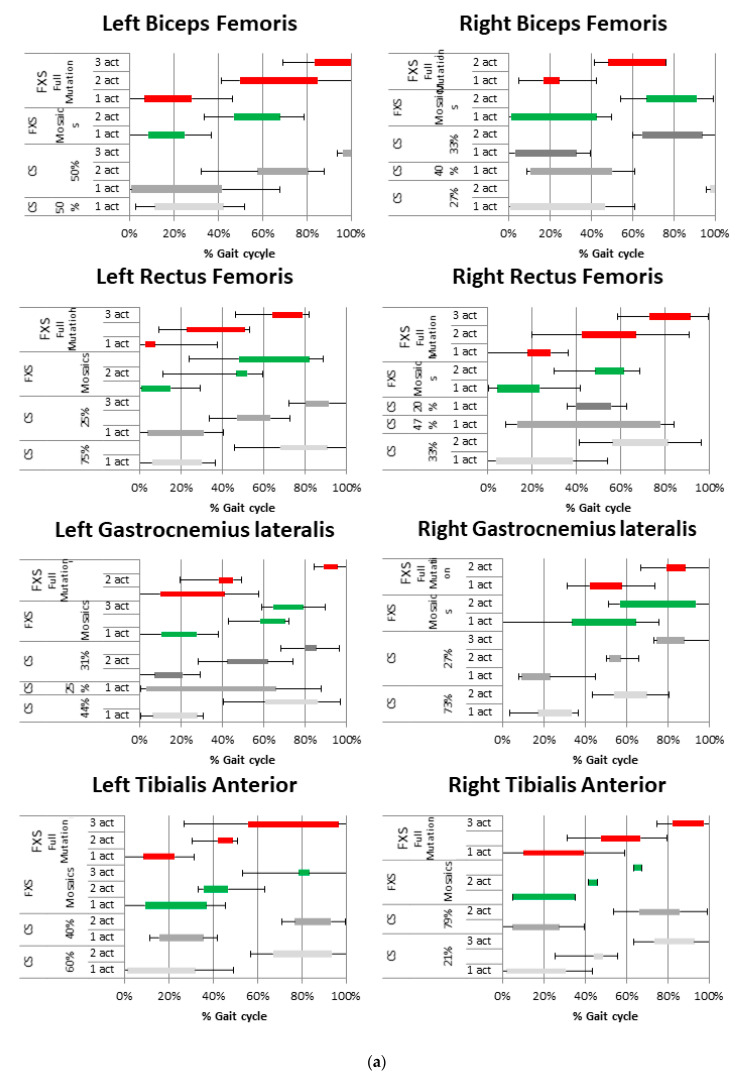

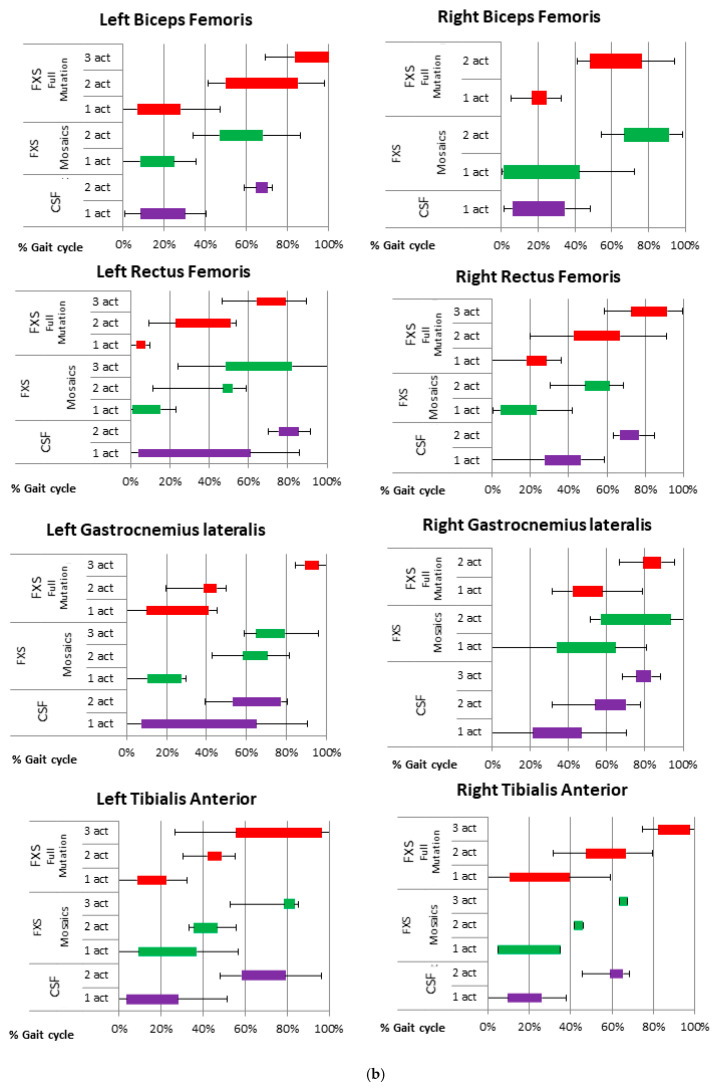

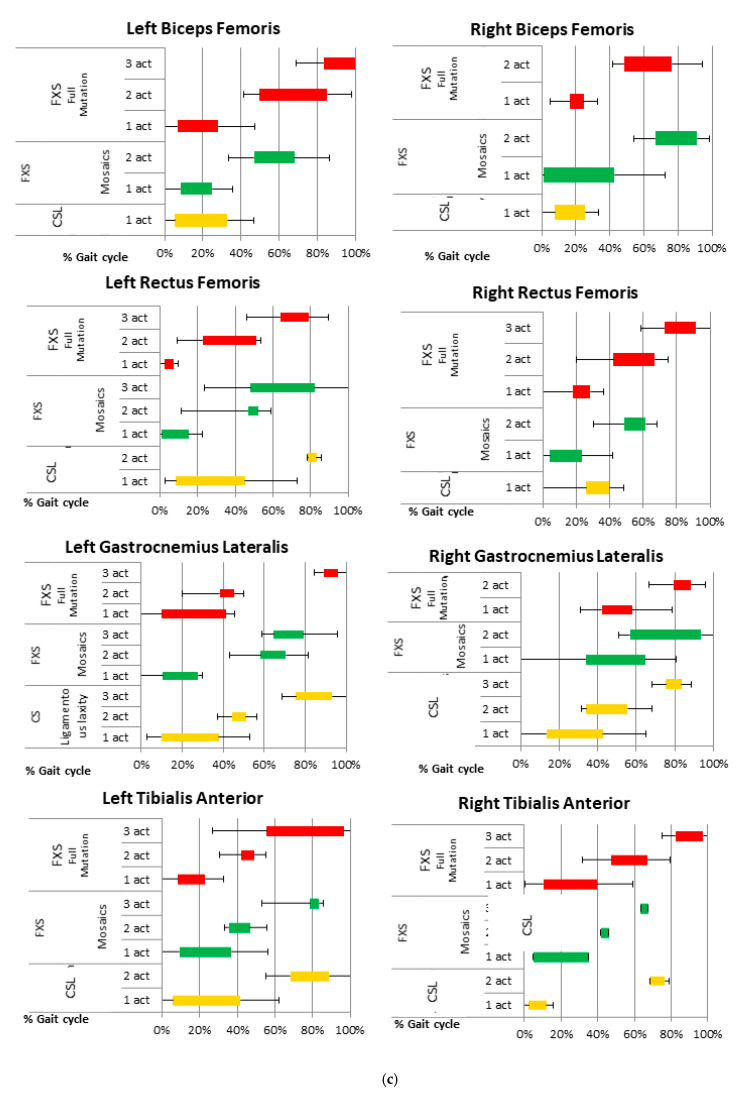
(**a**–**c**) Histograms presenting intervals of muscle activation during gait in FXS subjects and CS (mean values and ±1 SD), bilaterally for the following muscles: Biceps Femoris, Rectus Femoris, Gastrocnemius Lateralis, Tibialis Anterior. FXS Full Mutation (red), FXS Mosaics (green) and CS (grey, Figure 5a), CSF (purple, Figure 5b), CSL (yellow, Figure 5c). Colours refer to the online version of the figure.

In terms of the duration of muscle activity, our data indicate a prolonged activity of the muscles of the anterior compartment of lower limbs in fully mutated FXS subjects in comparison with mosaic individuals (see Figure 5).

**Figure 6:**
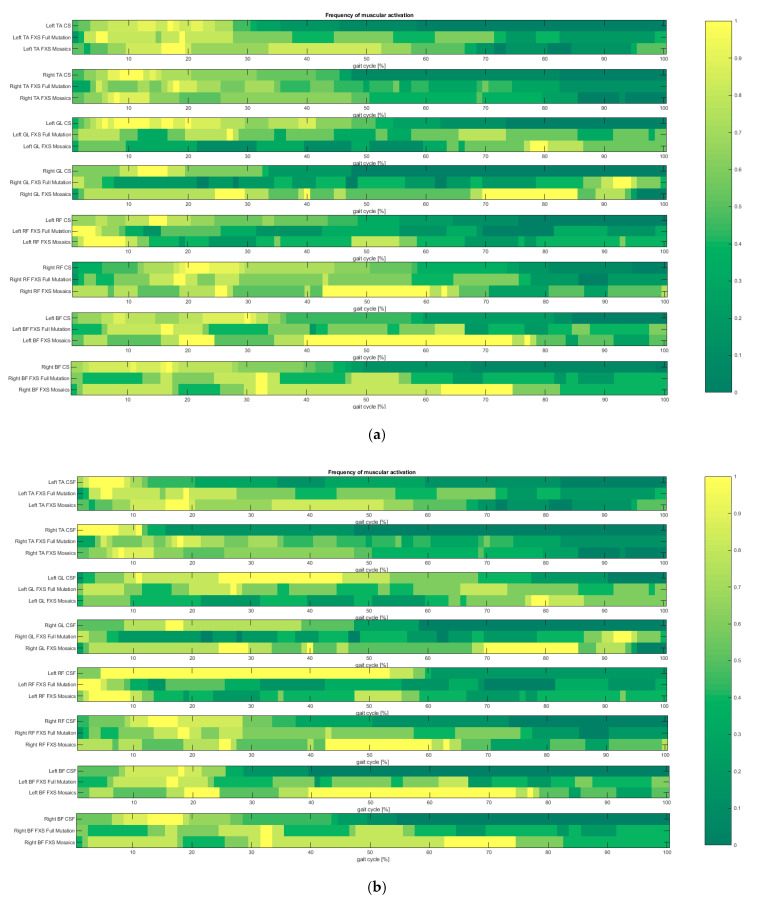

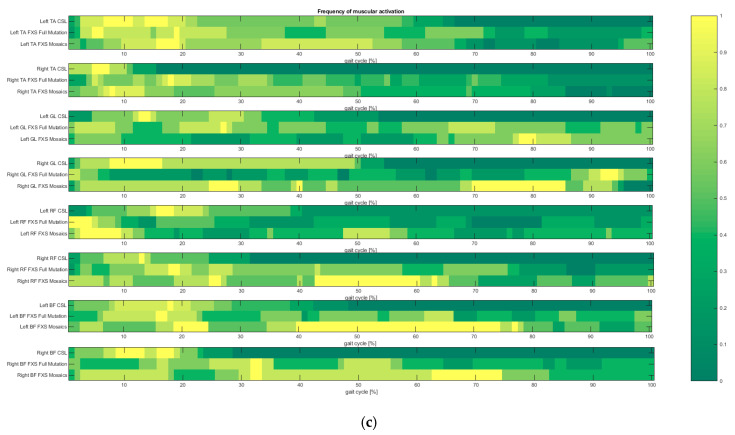
Frequency of muscle activation in analysed muscles in FXS Full Mutation, FXS Mosaics and CS (**a**), CSF (**b**) and CSL (**c**); horizontal bars are colour coded according to the number of subjects in which a muscle activity at each percentage of gait cycle is observed; yellow: muscle activity is detected in all subjects, dark green: muscle is not detected in any subject. Colours refer to the online version of the figure.

A higher frequency of muscle activations in both FXS groups was detected during both pre-swing and swing phases in all analysed muscles. Additionally, the FXS Full Mutation group displayed a pattern of multiple short activations detected throughout the entire gait cycle (see Figure 6).

In terms of the peak of the envelope, the highest value was detected in the FXS Full Mutation group (Table 3, Supplementary Materials). A delay in the position of the peak was highlighted in both FXS Full Mutation and FXS Mosaics groups in the Biceps Femoris, Gastrocnemius Lateralis, left Tibialis Anterior and left Rectus Femoris. 

## 4. Discussion

The most important findings of this study are: (1) The lower limb sagittal angles estimated through video-based motion analysis were deemed reliable and appropriate to be used with confidence in clinical settings for assessing the gait of FXS children;

(2) These variables, coupled with sEMG, allowed identification of a characteristic gait pattern specifically associated with this syndrome.

These results highlight previously overlooked clinically actionable features of FXS, the most frequent cause of familial ID and the single most frequent monogenic cause of autism spectrum disorders. Although the motor deficits that define the FX-associated phenotype of FXTAS have been studied [44], this is a disorder in which pathogenic mechanisms are completely different from those responsible for FXS.

FXS, in fact, is also characterized by locomotor alterations associated with hypotonia, ligamentous laxity and flat foot, which have never been fully investigated. On the other hand, locomotor disorders in several childhood neurological conditions, and now even in children with neurodevelopmental disorders with ID, have been assessed in the context of gait analysis [5,45,46,47,48,49,50].

Children with FXS suffer from a neurodevelopmental condition in which ID is associated with a high level of social anxiety, hyperactivity and sensory hypersensitivity. The combination of these clinical features severely hampers the possible use of routine gait analysis in a dedicated laboratory, with stereophotogrammetric systems, requiring the application of retroreflective markers on specific anatomical landmarks.

Overcoming these difficulties was a challenge for our work. We, therefore, as a proof of concept, verified the feasibility of gait analysis within ambulatory conditions, in an environment that FXS children could recognize as friendly, without the need for a dedicated laboratory. Hence, we used a gait analysis technique that, by avoiding the use of retroreflective markers, would be, and would be perceived as, less invasive. In order to detect signs of altered muscle activity we applied four sEMG probes to the children’s lower limbs. This particular setting, and the conditions in which the gait analysis was performed, were key elements for the very good compliance we obtained from all the enrolled children.

Reliability and repeatability of the markerless technique for assessing 2D lower limb kinematics was assessed in four different conditions in order to highlight the specific role of possible extrinsic sources of variability. The results were in line with previous literature reports [33,34]. Since joint angles are computed based on the bone embedded frames defined on anatomical landmarks’ trajectories, the different sources of variability were analysed, taking into account on one side the anatomical landmarks’ trajectories (i.e., comparison between set ups 1, 2, 3 and 4) and on the other side, the impact of a simplified joint embedded frame definition (comparison between set ups 1 and 3).

In terms of joint rotation angles, the normative bands, defined with the data of age and BMI matched healthy controls, find agreement with the corresponding bands reported in Leardini et al. 2007 [14], who adopted a stereophotogrammetric system. Our results showed that hip, knee and ankle joint rotations computed with a reduced marker set were comparable with that retrieved with a more complex marker set [14] on the sagittal plane.

Although video motion analysis is routinely performed by prosthetists and orthotists mainly using visual observation [51], recent literature reported encouraging results of a few applications of video-based 2D gait analysis in children with cerebral palsy [52] and Parkinson Disease’s adults [12].

In terms of comparisons between FXS children and CS, our study revealed a consistently altered pattern in terms of both kinematic parameters and muscle activity. In detail, joint kinematics highlighted an excessive hip and ankle flexion and, in contrast, a reduced knee flexion all over the gait cycle. As far as spatiotemporal parameters, longer stance period, reduced swing phase duration, cadence and gait speed are characteristic of both Full Mutation and Mosaic FXS individuals, a reduced stride length was specifically observed in the Full Mutation subjects. All these characteristics define a less stable gait; similar kinematics findings were reported in subjects with different neurodevelopmental disorders or neurological conditions associated with ID [5]. In particular, individuals with Down Syndrome showed a reduced range of motion in the sagittal plane at both hip and knee joints accompanied by lower ankle angles both in dorsiflexion and in plantarflexion and a higher hip flexion, compensated for by a reduced knee flexion angle during the swing phase of gait. Subjects with Prader –Willi syndrome displayed a gait characterized by reduced range of motion at the ankle joint but with excessive knee flexion, an increased period of stance in %, a reduced speed (but greater than those with Down Syndrome) and a shorter stride length [7].

In subjects with Ehlers–Danlos syndrome, lower dorsi flexion both in stance and in the swing phase was detected. Finally, in children with Dravet Syndrome—a genetically heterogeneous neurological condition mainly characterized by early-onset epilepsy—an increased ankle, knee and hip flexion during stance, reduced walking speed and stride length were observed.

As far as the effect of joint laxity and flat foot in the above mentioned populations, a dysfunctional walk was observed in association with a more flattened arch responsible for a lower capacity of generating power at the ankle joint during the push-off. In this respect, the authors suggested a specific walking rehabilitation protocol aimed at improving and increasing the strength of the foot muscles from the early stages of growth to counteract the effects of hypotonia and joint laxity with more efficacy [53].

Taken together, the data we generated seem to suggest that neither a flat foot nor a condition of ligamentous laxity can explain the observed motor alterations.

The identification of a seemingly specific FXS motor pattern, and its interpretation as a an effect likely related to intellectual disability, definitely increases the importance of investigating the relationships between cognition and the motor control of gait. This said, our study significantly differs from previous reports that were centred on clinical conditions without a precise genetic aetiology. Our data, on the contrary, were derived from a cohort of individuals affected with a well-known and truly monogenic condition.

In our small but well characterized cohort of individuals with classical FMR1 full mutations, the above-mentioned kinematic alterations were accompanied by altered activity of the muscles of the anterior and posterior aspects of the lower limbs. On the other hand, mosaic subjects presented similar but attenuated characteristics, consistent with that expected from the phenotypic modulatory effect of somatic mosaicism.

The observed alterations, represented by an increased number of activation and deactivation phases, seem to be a compensatory mechanism aimed at coping with the reduced range of motion registered at the lower limbs. It should be further mentioned that an asymmetric pattern was observed both in terms of activation and deactivation phases on the Gastrocnemius Lateralis and the Biceps Femoris, and in terms of the peak of the envelope, on the Tibialis Anterior and on the Biceps Femoris.

Muscle compensatory mechanisms could be due to the hypotonia or to the ligamentous laxity, two well-documented FXS clinical features likely originating in the alteration of the connective tissue that characterizes this condition [54]; however, it is worth noticing that in our controls with either of these features, we did not observe the same muscle alterations (see Figure 3 and Figure 4).

This indicates that all FSX subjects do adopt a common and peculiar muscle activity, a strategy that seems to be specific. On the other hand, it is not possible to exclude that a further explanation for a specific compensatory mechanism in FXS could be lying in the problem of balance impairment highlighted in our cohort by the spatiotemporal data. Indeed, previous investigations have demonstrated an association between reduced gait speed, cadence, and step length and balance impairments in subjects with vestibular disease [55]. To the best of our knowledge, specific problems of balance impairment have only been documented in older carriers of FMR1 permutations, affected by the neurodegenerative associated phenotype, FXTAS [23,24,56,57]. Due to the complexity of balance evaluations, which require standing still on a force plate for at least 40 s in eyes open and eyes closed conditions [23,24,56,57], these tests were not performed in the children of our cohort, all of whom had hyperactivity.

As previously mentioned, FXS mosaic individuals were found to have a muscle activity pattern much closer to that of controls. This observation could indeed be explained by the important phenotype modulation that a genetic mosaicism can exert. The same degree of compensation seen in subjects who carry a classical FMR1 full mutation might not be needed in FXS mosaic individuals because of a milder muscle alteration or possibly higher cognitive function, one of the crucial determinants of gait [5]. One could anticipate that, at a later age, some of these subjects could experience further muscle and balance alterations due to the presence of a concurrent premutation; the limits of current scientific knowledge on the risks of developing FXTAS and the young age of our cohort do not allow us to infer this information [23,24,54,56].

In agreement with previous findings [5], our data strengthen the hypothesis that a characteristic pattern of hyperflexed hip and ankle joints with hyperextended knees suggests the presence of an overall immature motor control of gait, and this might be the hallmark of gait in these individuals. The results of this study could be used to tailor specific rehabilitation protocols that aim to restore a more efficient gait pattern. This agrees with the literature [57] suggesting the adoption of specific walking re-education protocols even in pathologies linked by the presence of joint laxity, such as Down and Ehlers–Danlos syndromes. Indeed, in subjects with Down Syndrome, the walking strategies adopted are different from those with Ehlers–Danlos, who would benefit from a program for muscle strengthening, while subjects with Down Syndrome would probably need a combined program of muscle strengthening followed by gait retraining to reacquire the correct walking pattern [57].

This work suffers from some limitations that should be acknowledged: first, we have so far examined a rather restricted cohort of individuals within which we distinguished subjects with classical full mutations from subjects with size mosaicism; it should be mentioned that each tested subject is represented by six gait analysis trials and our statistics, which adopted a non-parametric test suitable for small subject groups, did not compare single individuals but rather number of gait trials.

We plan to expand the studied cohort in both mutational categories, in order to confirm and strengthen our results. Both control groups with ligamentous laxity and flat foot were small and should be increased in order to better distinguish the role that these orthopaedic features may play in the gait of FXS children. Furthermore, the gait analysis was performed out of a formal gait laboratory without the aid of a stereophotogrammetric system; by considering the impact of video based motion analysis on the precision of kinematic data, we only included the sagittal plane kinematics, which was found to be consistent with the gait profile generated by the stereophotogrammetric system [34,52]. Finally, in terms of muscle activity, our data displayed a large variability in terms of envelope peak, and this finds agreement with Granata et al. [58], who reported large variability in terms of both envelope and on-off timing of muscle recruitment in children.

## 5. Conclusions

The gait analysis we report was not performed in standard laboratory conditions, and without the precision of stereophotogrammetry; we nevertheless believe this study for the first time provides an objective measure of the motor control of gait, a clinical feature never previously studied in FXS. This measure could be clinically actionable and could represent an additional tool in the assessment of new pharmacological treatments for this specific monogenic neurodevelopmental disorder. This would be crucial, since the lack of robust and reliable clinical biomarkers and of a quantitatively measurable outcome have substantially limited the success of FXS pharmacological trials [59,60], despite different functional pathways and mechanisms having been explored [23,54]. New ways to link molecular and physiological disease mechanisms to behavioural features are needed, and the current study might offer a possible contribution.

The application of clinical gait analysis to our FXS cohort, small because we are dealing with a rare disorder and decided to consider a limited developmental age range, has nevertheless allowed us to identify characteristic gait deviations. Although preliminary, the results of our study provide evidence of a relationship between physical characteristics and gait features in subjects with FXS.

We demonstrate how the study of gait analysis, performed within routine clinical assessments, represents an effective tool for monitoring the motor control of gait in children and adolescents with FXS, and can become a measurable outcome of physical and cognitive improvements, thus providing useful data for intervention planning.

## Figures and Tables

**Table 1:** Demographic characteristics of the studied subjects: 12 FXS children, of whom 7 carried a classical full mutation of the FMR1 gene (FXS Full Mutation) and 5 a full mutation with mosaicism (FXS Mosaics); 10 controls without any foot deformities or presence of ligamentous laxity (CS), 6 controls with a flat foot (CSF) and 3 controls with ligamentous laxity (CSL).

	No. of Subjects	Male/Female	Age [Years] ± SD	Body Mass [kg] ± SD	Body Height [m] ± SD	BMI [kg/m^2^] ± SD
FXS Full Mutation	7	0/7	9.57 ± 2.51	35.4 ± 14.5	1.34 ± 0.12	19.0 ± 5.54
FXS Mosaics	5	0/5	9.00 ± 3.74	34.8 ± 19.1	1.32 ± 0.25	18.7 ± 2.61
CS	10	1/9	9.55 ± 2.79	35.1 ± 9.49	1.39 ± 0.16	20.7 ± 4.67
CSF	6	0/6	11.4 ± 1.80	49.7 ± 13.3	1.50 ± 0.10	23.9 ± 3.25
CSL	3	0/3	11.0 ± 3.60	41.3 ± 12.5	1.40 ± 0.21	19.4 ± 0.95

**Table 2:** Comparison of RMSD and RMSD in % of the Gold Standard, in terms of mean and standard deviation (SD), for each set up and for each joint angle, with those assessed by [38,39,40].

**RMSD Mean (SD)**	**GS vs. Marker**	**GS vs. Tape**	**GS vs. No Tape**	**Marker vs. Tape**	**Marker vs.** **No Tape**	**Tape vs. No Tape**	**Castelli 2014 [39]** **—Gait Speed Normal**	**Castelli 2015 [40]** **—Comfortable**	**Ceseracciu 2014 [38]**
Hip	1.83 (1.23)	2.59 (1.37)	2.44 (1.39)	0.88 (0.38)	4.17 (2.08)	2.96 (1.72)	2.3	4.8	17.6 (8.5)
Knee	3.38 (1.75)	4.29 (2.35)	3.51 (2.48)	2.57 (1.64)	2.12 (1.85)	2.21 (2.00)	2.44	3.6	11.8 (2.5)
Ankle	2.58 (1.72)	4.73 (2.14)	2.38 (2.05)	2.91 (0.98)	8.77 (1.65)	3.67 (2.05)	3.53	3	7.2 (1.8)
**RMSD% Mean**	**GS vs. Marker**	**GS vs. Tape**	**GS vs. No Tape**	**Marker vs. Tape**	**Marker vs.** **No Tape**	**Tape vs. No Tape**	**Castelli 2014 [39]** **—Gait Speed Normal**	**Castelli 2015 [40]** **—Comfortable**	**Ceseracciu 2014 [38]**
Hip	3.22	4.55	4.29	1.54	6.04	5.21	4	/	44.7
Knee	4.57	5.81	4.74	3.48	2.15	2.99	3	/	18.3
Ankle	5.07	9.31	6.48	5.72	14.3	7.22	4	/	33.1

**Table 3:** Comparison of the value of the peak of the envelope [% of mean value] and position of the peak of the envelope [% of gait cycle], in terms of median and inter quartile range (IQR), for FXS Full Mutation, FXS Mosaics, CS, CSF and CSL. Statistically significant differences (*p*-value of the Wilcoxon rank-sum test >0.05) are reported with * with respect to the FXS Full Mutation, ** with respect to the FXS Mosaics, *** with respect to the CS, **** with respect to CSF, ***** with respect to CSL.

**Normalized Peak of the Envelope Median (IQR)**	**Left TA**	**Right TA**	**Left GL**	**Right GL**	**Left RF**	**Right RF**	**Left BF**	**Right BF**
FXS Full Mutation	260.84 (108.29) *** ****	254.28 (91.37) ** ****	333.99 (139.47) *** **** *****	319.19 (152.85) ****	273.22 (185.08) ** ****	244.97 (159.18) *** ****	268.94 (151.99) ** *** ****	322.27 (141.50) **
FXS Mosaics	249.27 (167.94) *** ****	309.39 (28.26) * *** ****	296.29 (157.44) *** **** *****	251.38 (63.47) ****	222.69 (69.48) * *** *****	223.29 (53.66) ****	229.77 (157.63) * ****	219.88 (118.33) * *** **** *****
CS	206.37 (97.75) * **	220.65 (121.82) ** *****	234.72 (107.11) * **	254.10 (154.23) ****	238.64 (49.87) ** ****	210.92 (117.49) * **** *****	267.34 (66.76) *	286.04 (76.23) **
CSF	206.84 (21.71) * **	213.77 (41.93) * ** *****	219.30 (51.13) * **	175.24 (69.04) * ** *** *****	183.06 (50.01) * *** *****	253. 50 (661.66) *** *****	217.06 (79.96) * ** *****	297.01 (117.31) **
CSL	233.77 (46.19)	308.95 (176.72) *** ****	229.73 (83.32) * **	209.34 (66.91) ****	275.27 (73.73) ** ****	337.24 (205.14) * ** *** *****	262.44 (133,03) ****	305.05 (44.33) **
**Position of the Peak of the Envelope (%Gait Cycle)** **Median (IQR)**	**Left TA**	**Right TA**	**Left GL**	**Right GL**	**Left RF**	**Right RF**	**Left BF**	**Right BF**
FXS Full Mutation	58.40 (49.05) ** **** *****	46.35 (55.66) ** **** *****	72.18 (38.75) *** **** *****	68.98 (43.36) *** **** *****	61.58 (33.87) *** **** *****	60.29 (54.47) ** *****	60.28 (54.61) *** **** *****	52.98 (43.47) *****
FXS Mosaics	64.83 (31.51) ** **** *****	27.06 (43.01) * ***	67.91 (46.47) *** *****	68.81 (20.37) *** **** *****	78.29 (34.56) *** **** *****	30.40 (14.98) * ***	80.23 (28.23) *** **** *****	51.04 (44.47) *****
CS	45.46 (39.10) * **	30.87 (34.93) ** *****	44.66 (42.10) * **	38.85 (23.26) * **	60.79 (40.71) * ** *****	57.11 (48.38) ** *****	26.74 (33.27) * **	29.37 (36.35)
CSF	31.82 (69.48) * **	14.18 (61.50) *	27.72 (65.13) *	23.81 (33.66) * **	29.79 (47.01) * ** *****	27.53 (35.61) *****	22.73 (52.96) * ** *****	58.44 (45.81) *****
CSL	15.32 (11.77) * **	11.26 (12.16) * ***	19.87 (31.11) * ***	24.90 (59.32) * **	19.64 (5.81) * ** *** ****	19.48 (36.62) * *** ****	21.95 (15.10) * ** ****	15.14 (7.98) * ** ****

## Data Availability

The data presented in this study are available on request from the corresponding author. The data are not publicly available due to restrictions e.g. privacy or ethical.

## Supplementary Materials

The following are available online at https://www.mdpi.com/article/10.3390/s21144746/s1.
