# Supplementary material for: Feasibility and Reliability Assessment of Video-Based Motion Analysis and Surface Electromyography in Children with Fragile X during Gait
Source: Sensors (Basel). 2021 Jul 12;21(14):4746. doi: 10.3390/s21144746 (PMC8309640; doi:10.3390/s21144746)
Supplement: Supplementary file 1 [file sensors-21-04746-s001.zip › Figure A2 Table A2b.pdf]

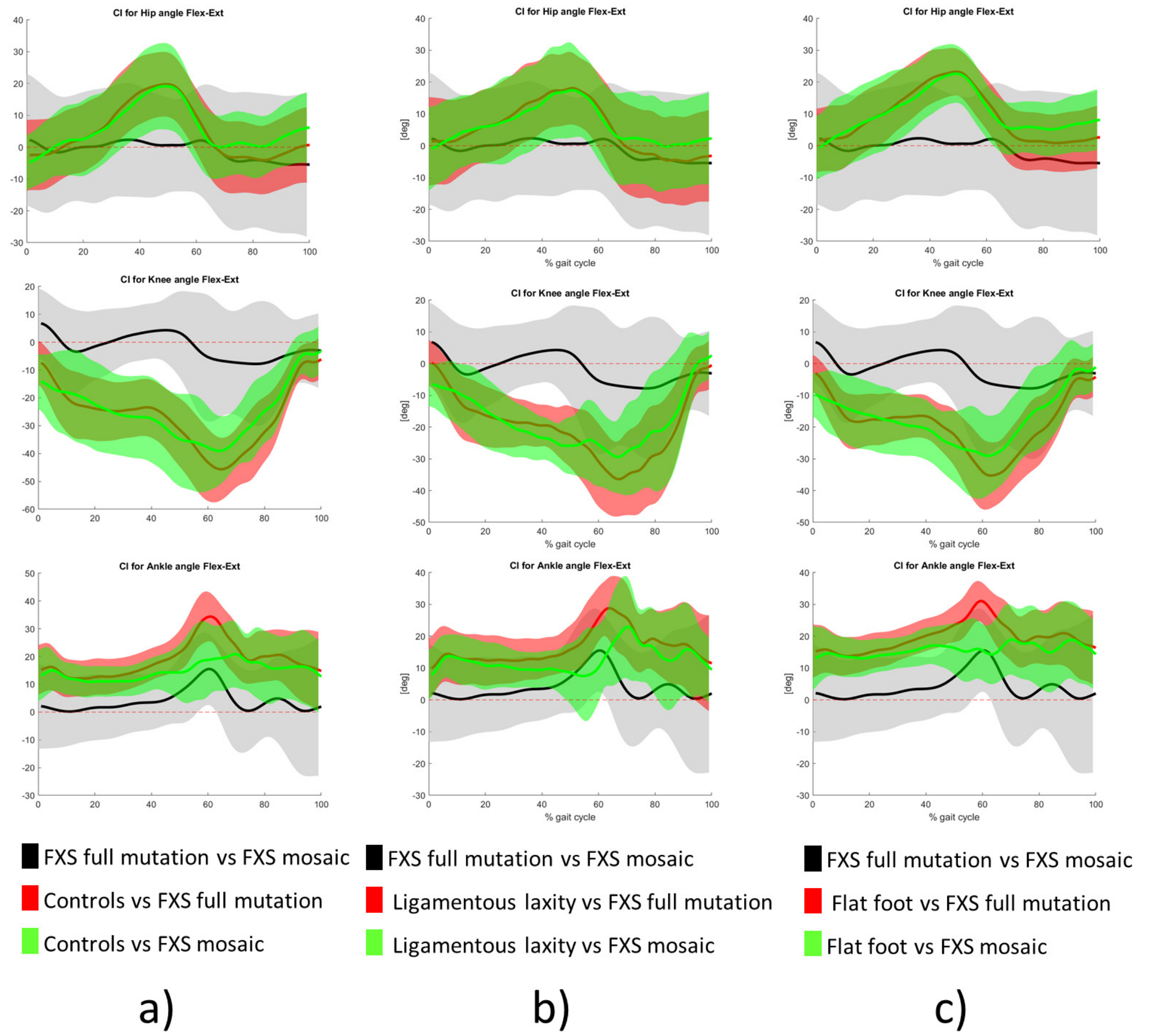

Figure A2a. Confidence intervals for the comparison of hip, knee and ankle sagittal plane angles between CS vs FXS Full Mutation vs FXS Mosaics (a), CSL vs FXS Full Mutation vs FXS Mosaics (b), CSF vs FXS Full Mutation vs FXS Mosaics (c)

Figure A2b. Confidence intervals and the difference between means of two compared groups for comparisons of the envelope profiles between FXS Full Mutation vs FXS Mosaics, CS vs FXS Full Mutation, CS vs FXS Mosaics are presented

FXS Full Mutation vs FXS Mosaics	CS vs FXS Full Mutation	CS vs FXS Mosaics
	Left Tibialis Anterior	
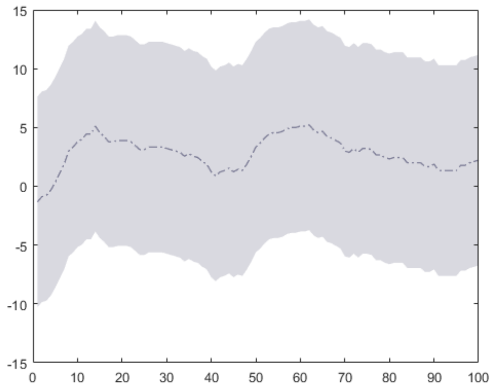	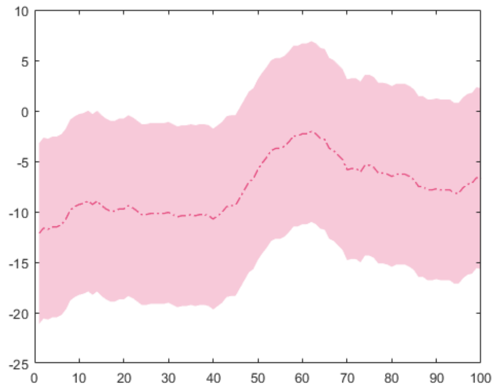	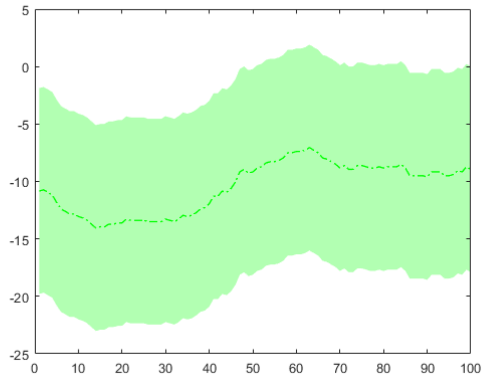
	Right Tibialis Anterior	
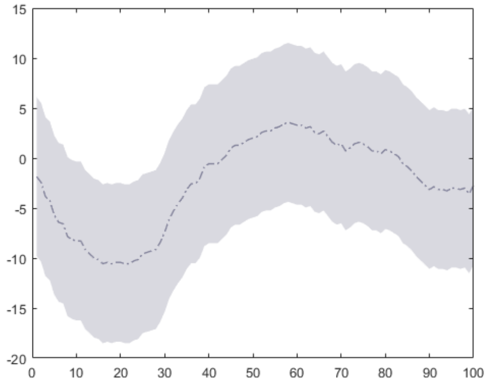	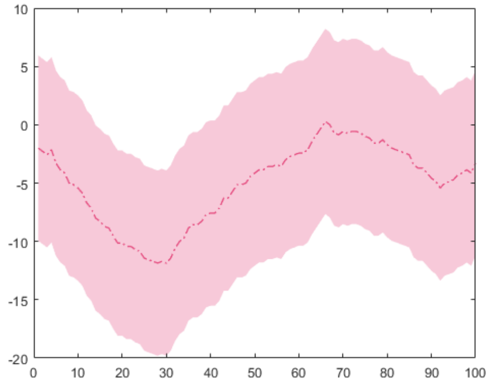	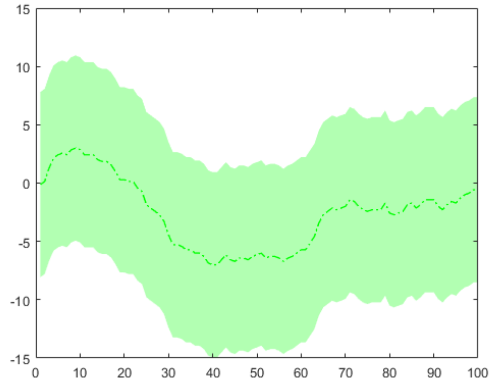
	Left Gastrocnemius Lateralis	

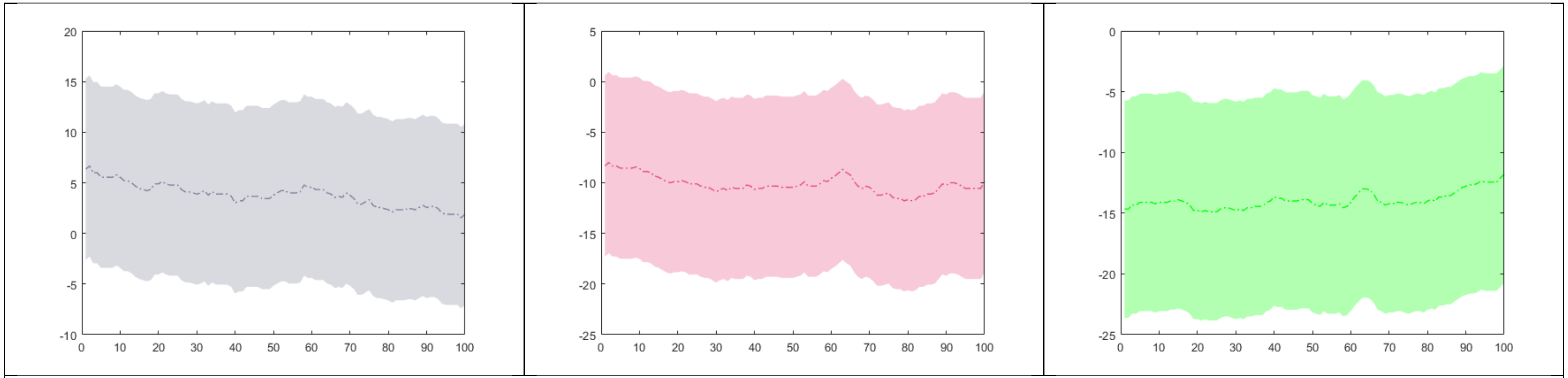

Right Gastrocnemius Lateralis

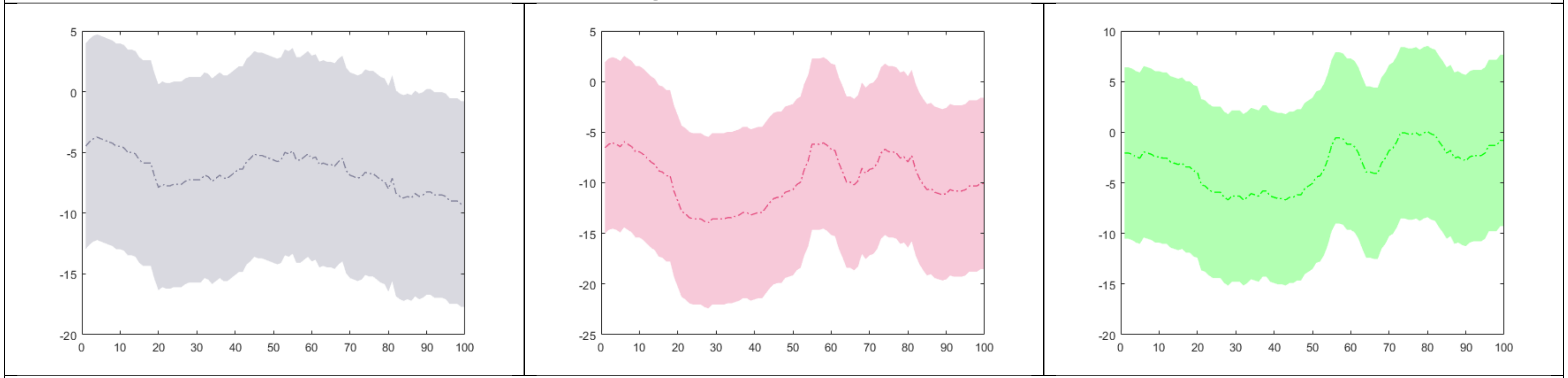

Left Rectus Femoris

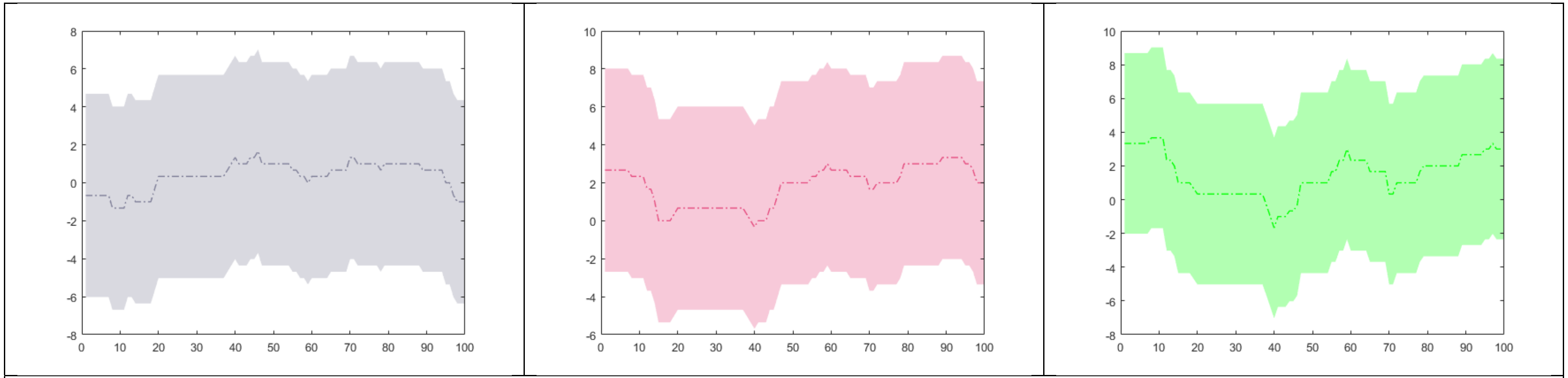

Right Rectus Femoris

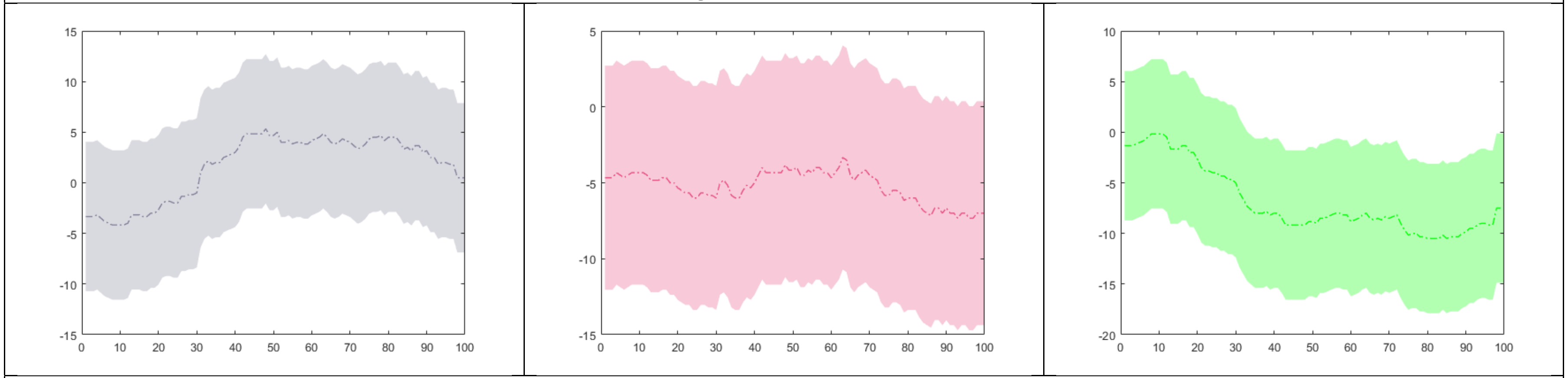

Left Biceps Femoris

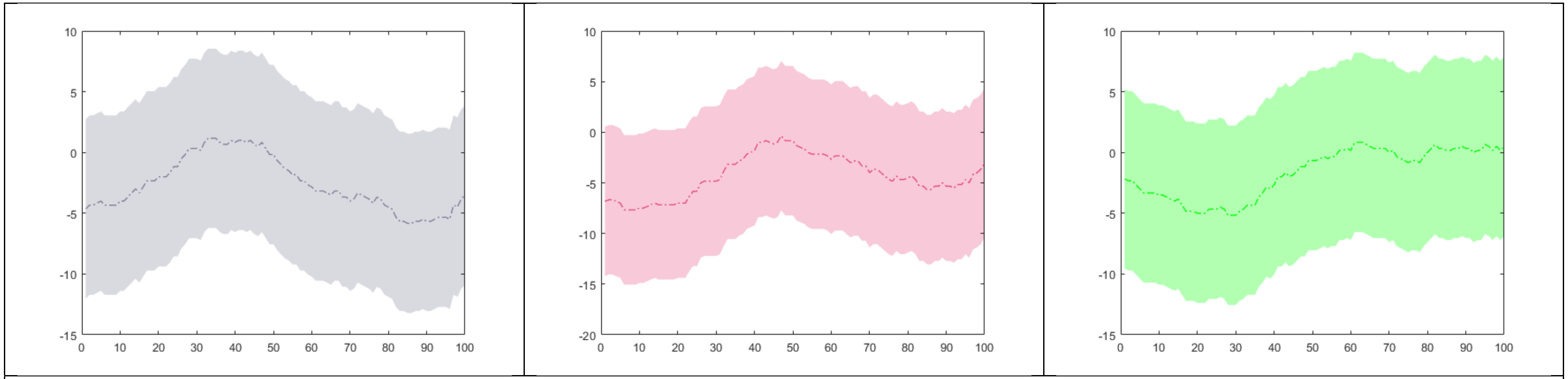

Right Biceps Femoris

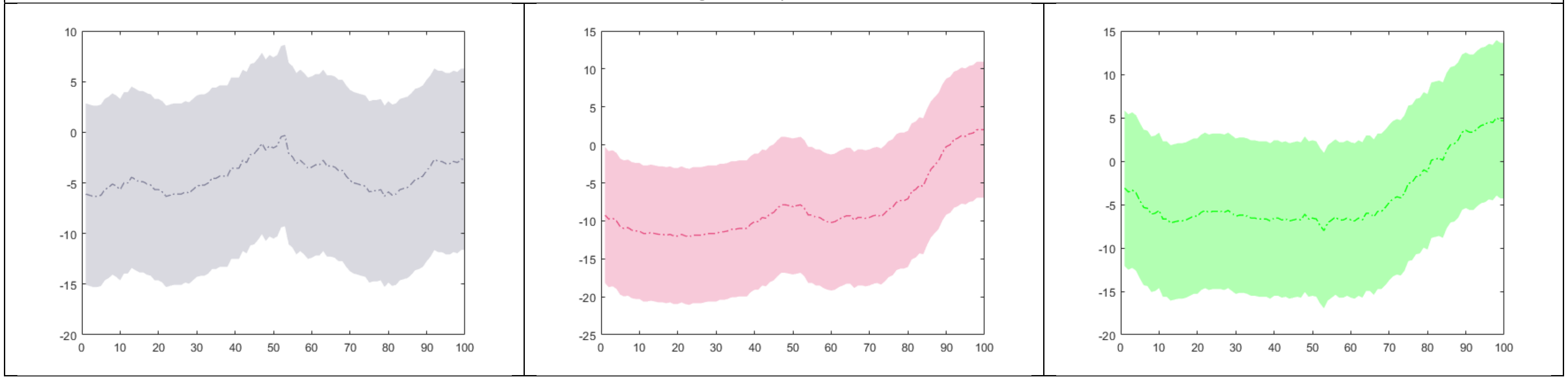

Figure A2c. Confidence intervals and the difference between means of two compared groups for comparisons of the envelope profiles between FXS Full Mutation vs FXS Mosaics, CSF vs FXS Full Mutation, CSF vs FXS Mosaics are presented.

FXS Full Mutation vs FXS Mosaics	CSF vs FXS Full Mutation	CSF vs FXS Mosaics
Left Tibialis Anterior		

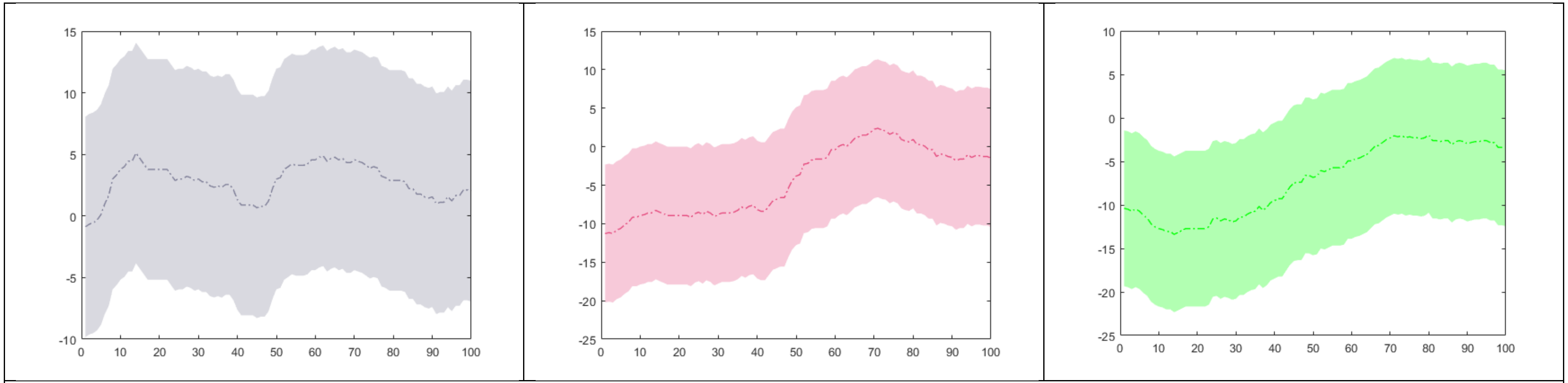

Right Tibialis Anterior

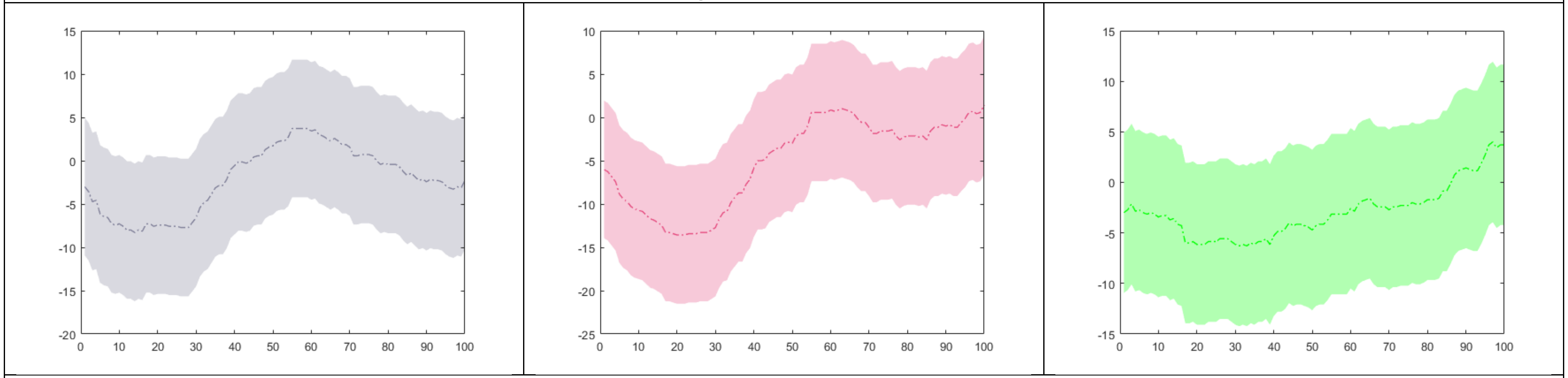

Left Gastrocnemius Lateralis

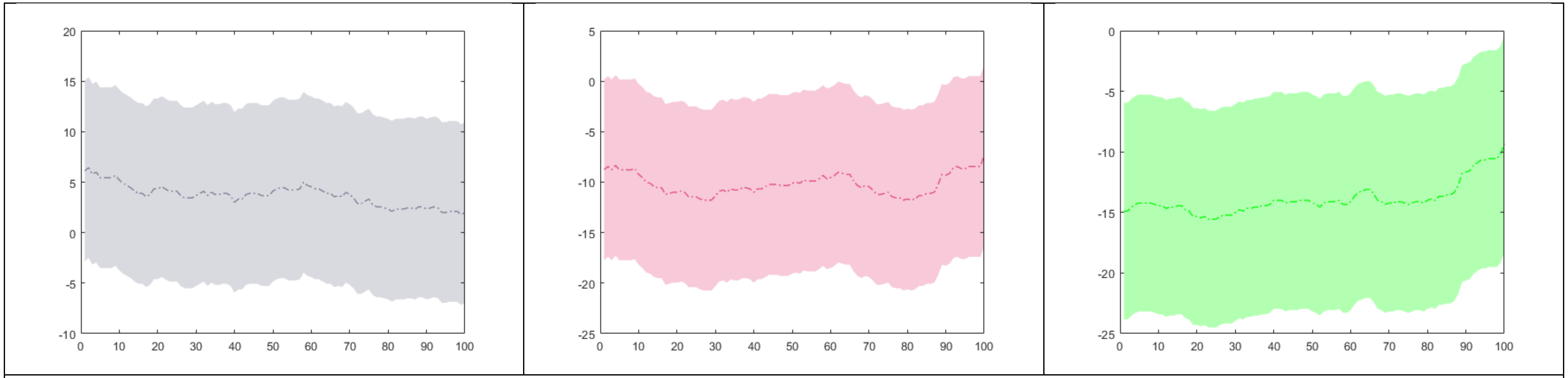

Right Gastrocnemius Lateralis

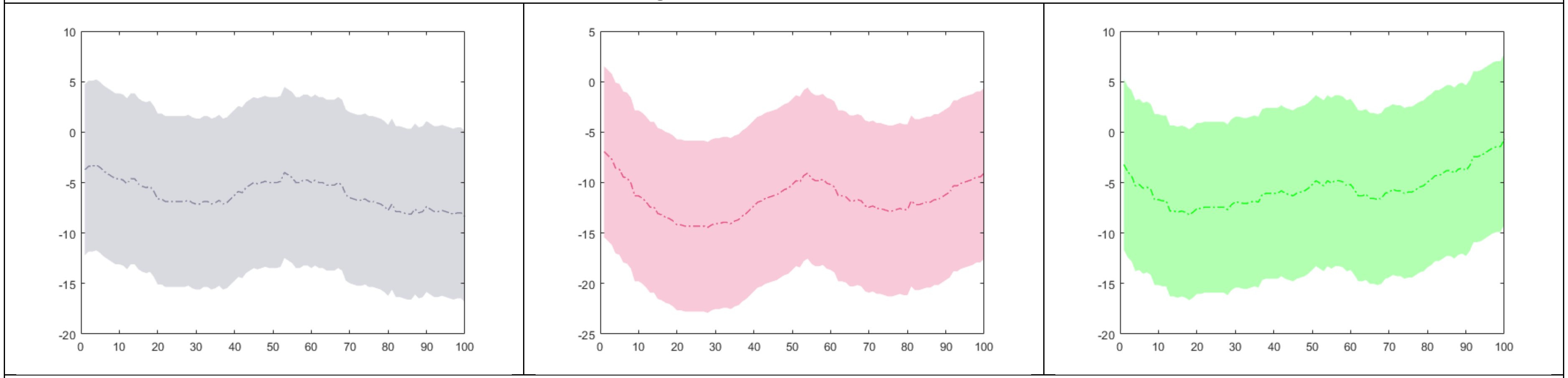

Left Rectus Femoris

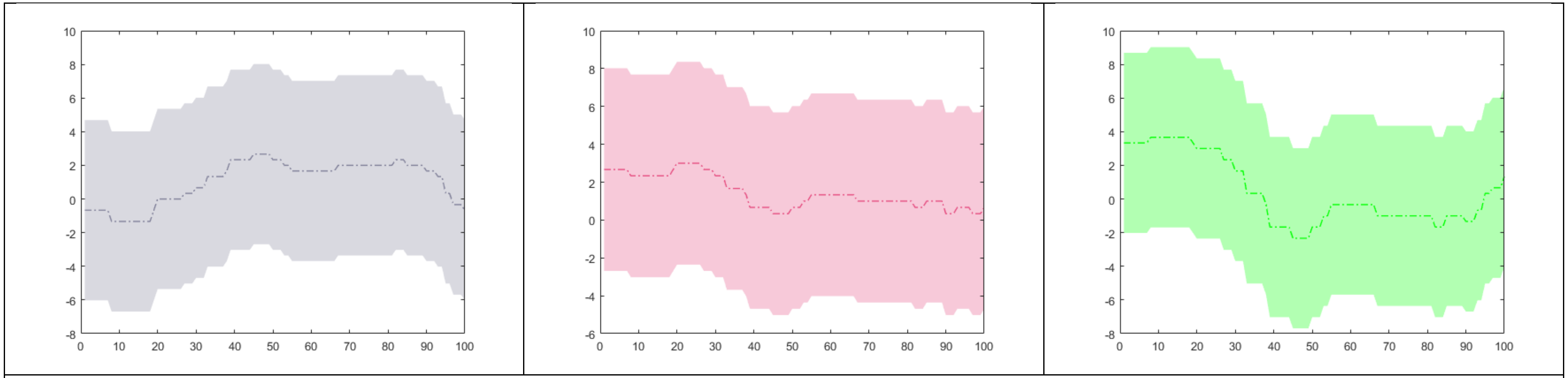

Right Rectus Femoris

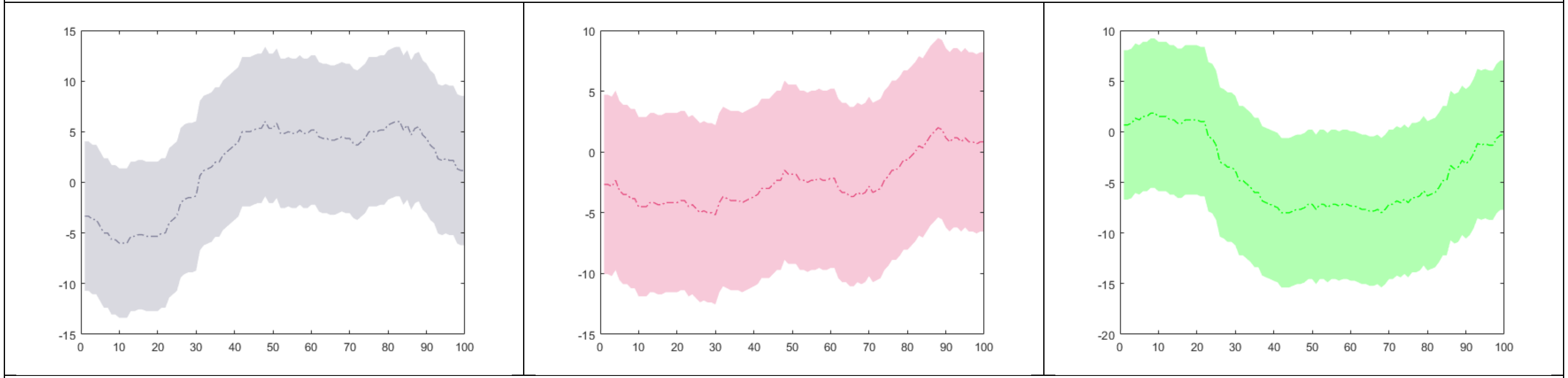

Left Biceps Femoris

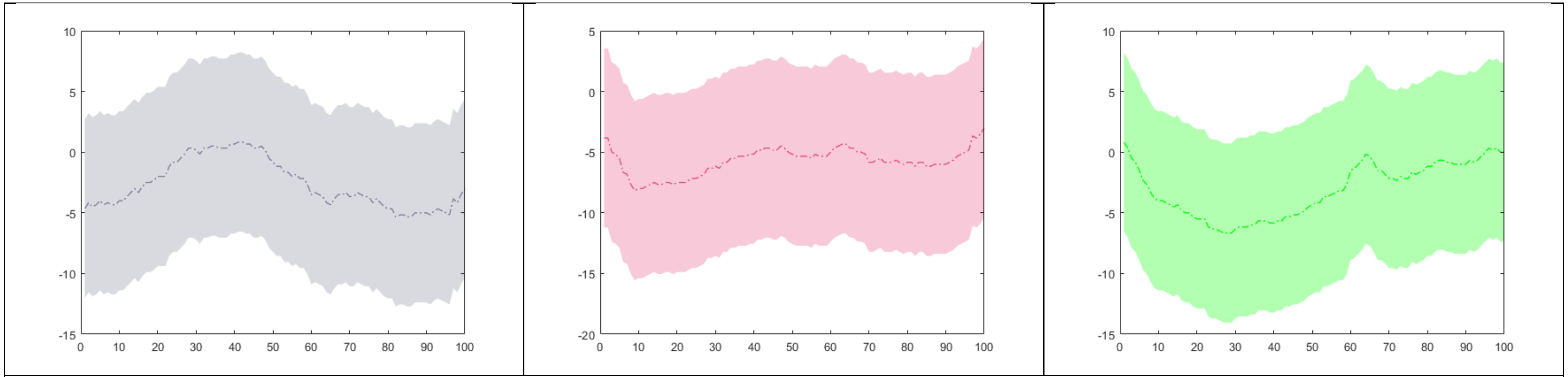

Right Biceps Femoris

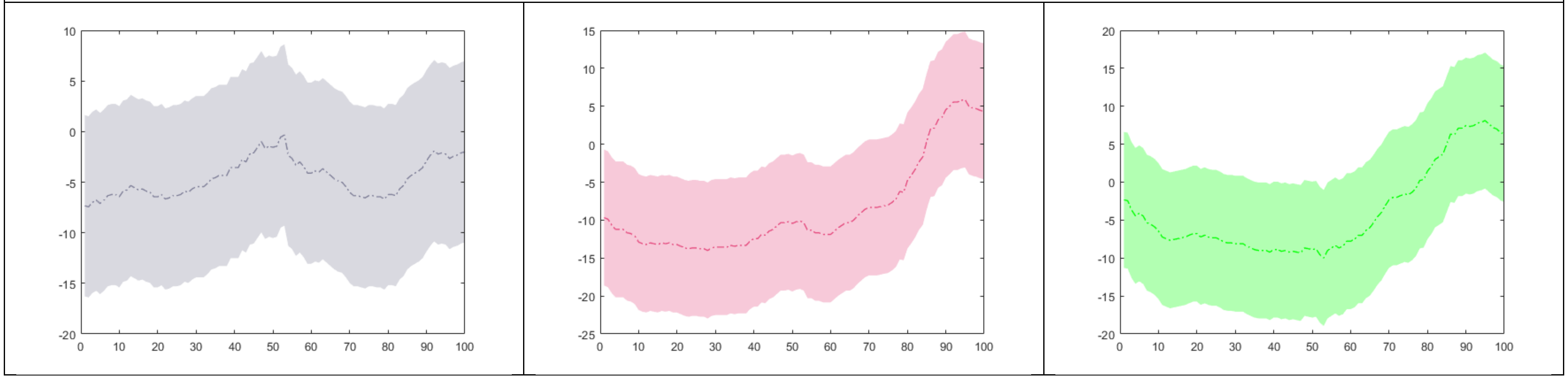

Figure A2d. Confidence intervals and the difference between means of two compared groups for comparisons of the envelope profiles between FXS Full Mutation vs FXS Mosaics, CSL vs FXS Full Mutation, CSL vs FXS Mosaics are presented

FXS Full Mutation vs FXS Mosaics	CSL vs FXS Full Mutation	CSL vs FXS Mosaics

Left Tibialis Anterior

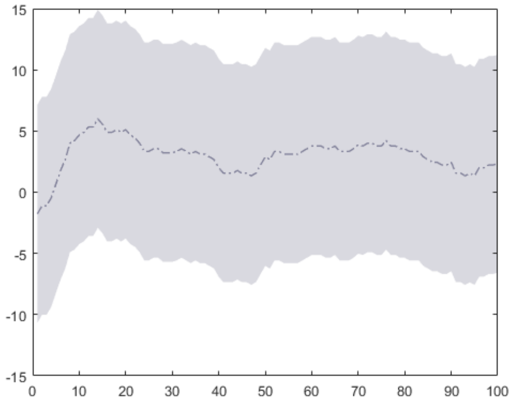

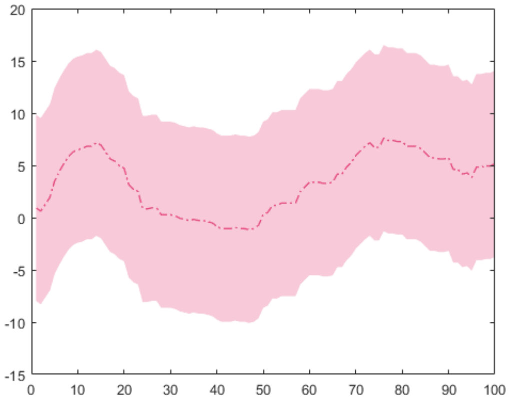

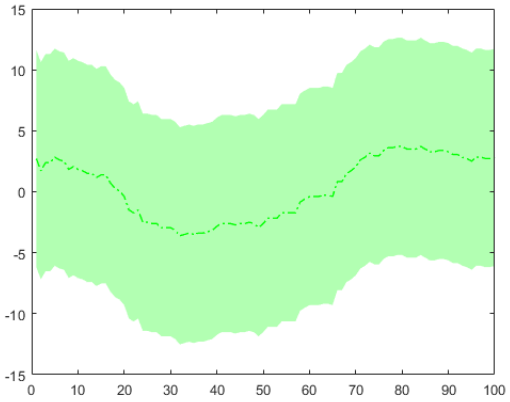

Right Tibialis Anterior

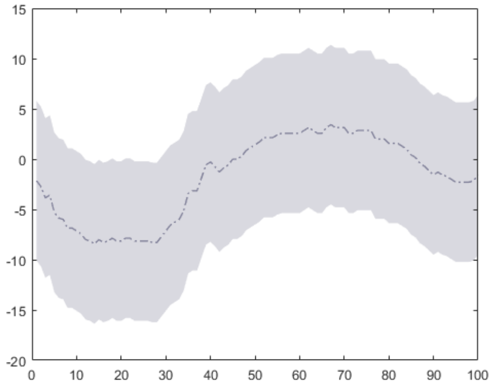

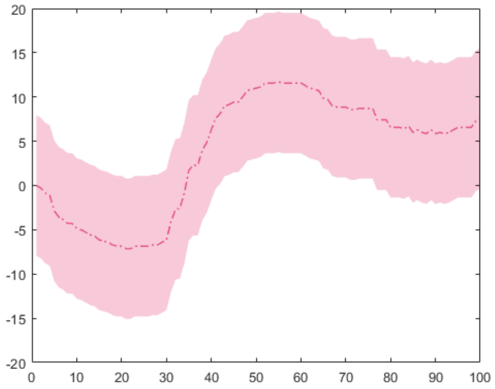

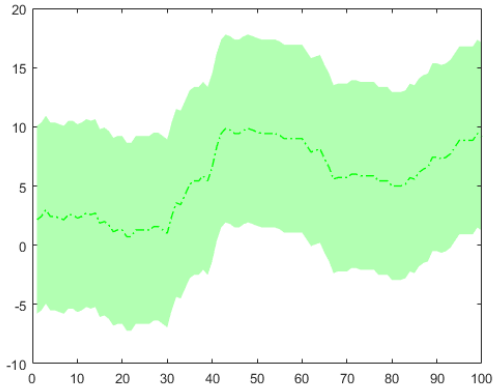

Left Gastrocnemius Lateralis

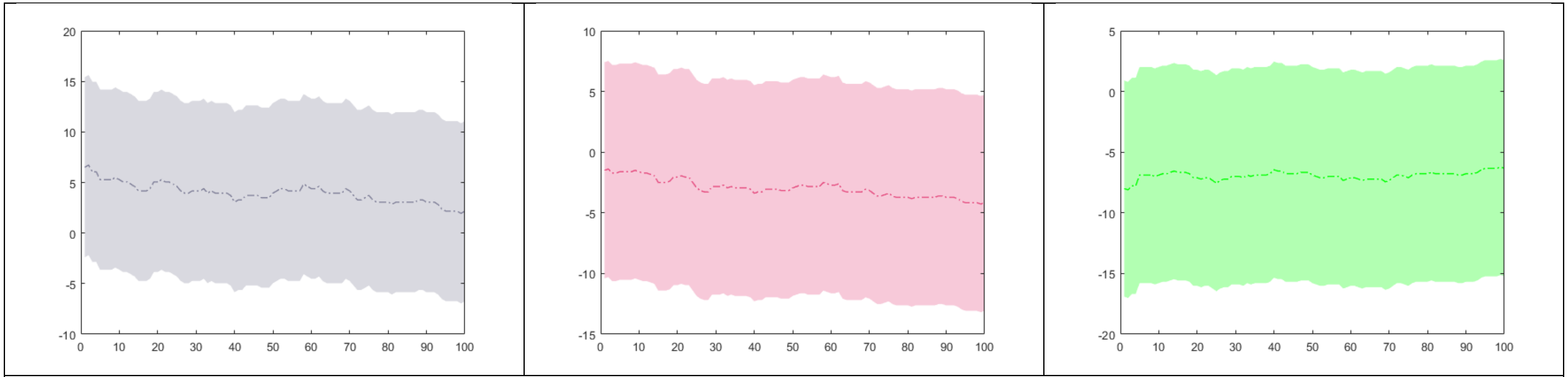

Right Gastrocnemius Lateralis

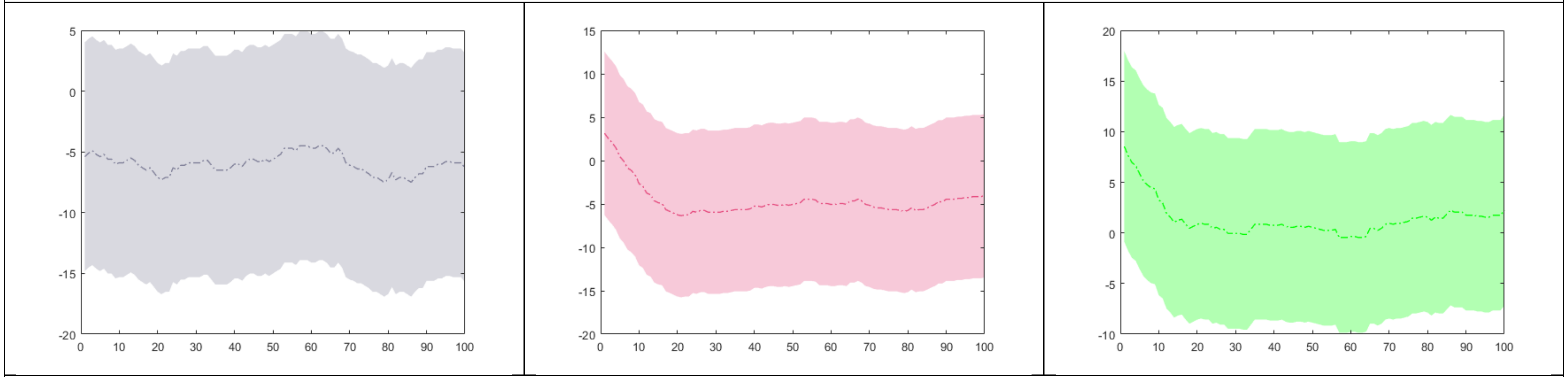

Left Rectus Femoris

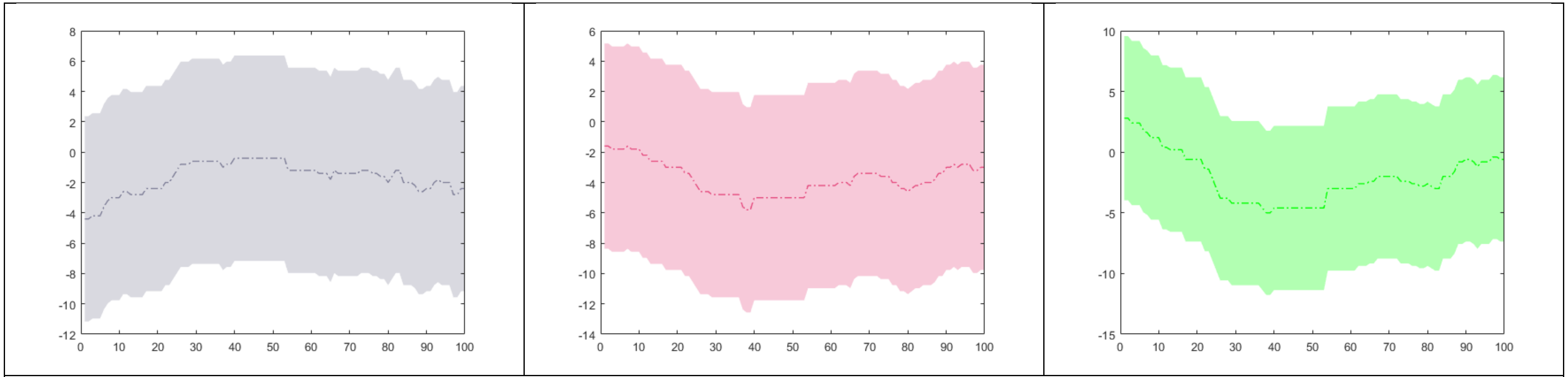

Right Rectus Femoris

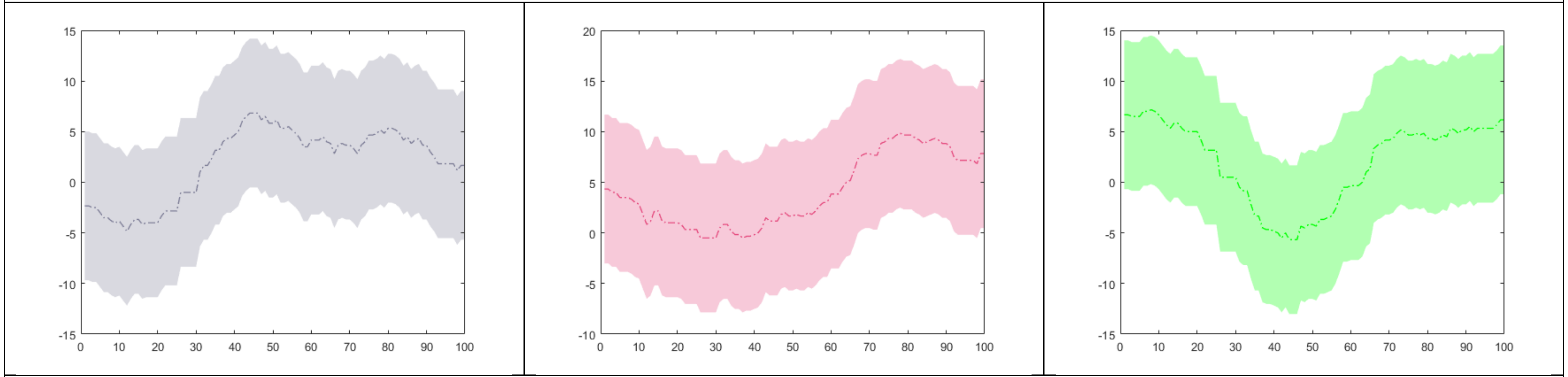

Left Biceps Femoris

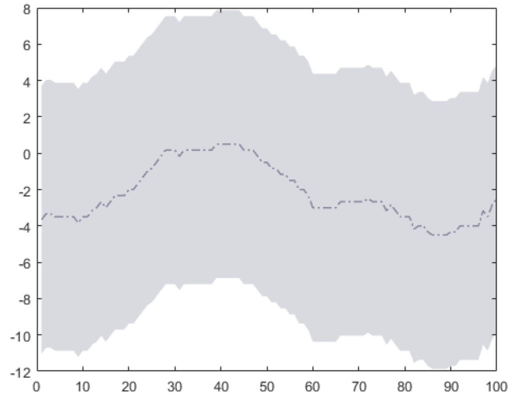

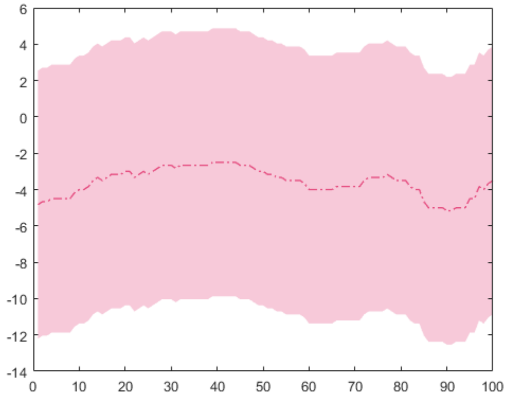

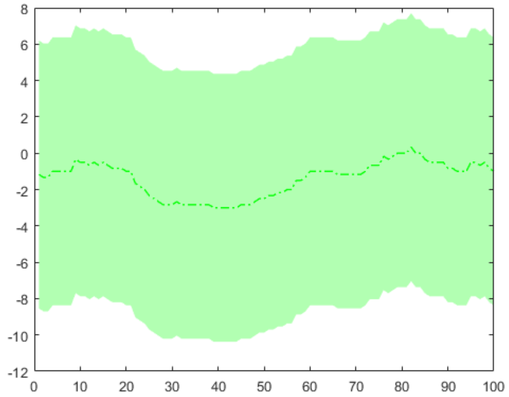

Right Biceps Femoris

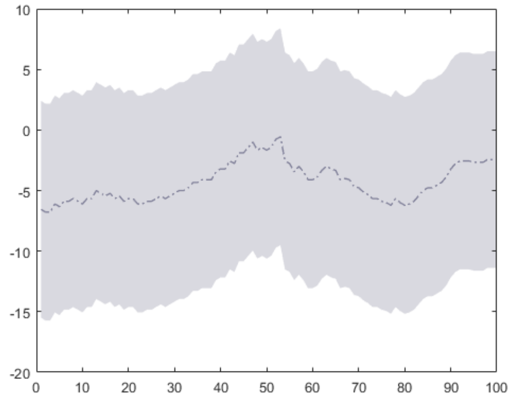

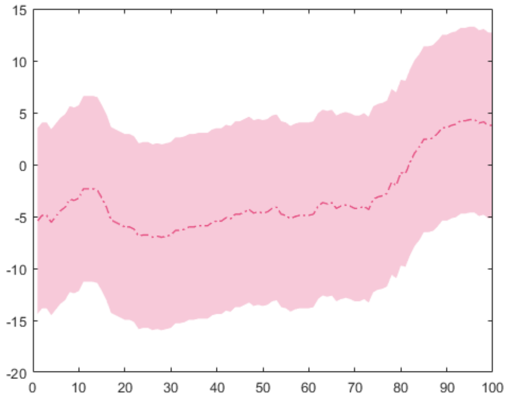

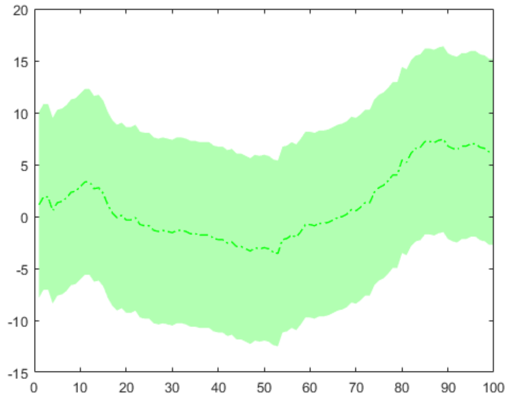

Table A2a. P value, lower and upper bounds of 95% confidence interval for the statistical analysis of spatio-temporal parameters

Parameter	Comparison	p	Lower bound of 95% confidence interval	Upper bound of 95% confidence interval
Stride length (m)	FXS Full Mutation vs FXS Mosaics	0.519	-0.237	0.076
	FXS Full Mutation vs CS	0.619	-0.032	0.215
	FXS Full Mutation vs CSF	0.037	-0.298	-0.085
	FXS Full Mutation vs CSL	0.013	-0.338	-0.048
	FXS Mosaics vs CS	0.566	-0.090	0.088
	FXS Mosaics vs CSF	0.074	-0.212	-0.005
	FXS Mosaics vs CSL	0.006	-0.013	0.214
	CS vs CSF	0.010	-0.171	-0.030
	CS vs CSL	0.028	-0.190	-0.005
	CSF vs CSL	0.567	-0.100	0.078
Stride time (s)	FXS Full Mutation vs FXS Mosaics	0.0.68	-0.033	0.233
	FXS Full Mutation vs CS	0.001	-0.300	-0.067
	FXS Full Mutation vs CSF	0.022	0.033	0.233
	FXS Full Mutation vs CSL	0.147	-0.067	0.200
	FXS Mosaics vs CS	0.001	-0.133	-0.033
	FXS Mosaics vs CSF	0.292	-0.033	0.100
	FXS Mosaics vs CSL	0.071	0.000	0.100
	CS vs CSF	0.051	-0.100	0.000
	CS vs CSL	0.000	-0.167	-0.100
	CSF vs CSL	0.021	0.033	0.133
Velocity (m/s)	FXS Full Mutation vs FXS Mosaics	0.561	0.382	0.073
	FXS Full Mutation vs CS	0.007	0.050	0.447

	FXS Full Mutation vs CSF	0.000	-0.502	-0.060
	FXS Full Mutation vs CSL	0.258	-0.404	0.027
	FXS Mosaics vs CS	0.090	-0.007	0.188
	FXS Mosaics vs CSF	0.009	-0.210	-0.037
	FXS Mosaics vs CSL	0.632	-0.079	0.168
	CS vs CSF	0.175	-0.086	0.017
	CS vs CSL	0.241	-0.030	0.163
	CSF vs CSL	0.032	-0.189	-0.013
Stance (%)	FXS Full Mutation vs FXS Mosaics	0.265	-4.074	2.051
	FXS Full Mutation vs CS	0.007	-4.598	-0.639
	FXS Full Mutation vs CSF	0.000	1.255	5.376
	FXS Full Mutation vs CSL	0.825	-1.347	3.333
	FXS Mosaics vs CS	0.000	-5.447	-2.098
	FXS Mosaics vs CSF	0.000	3.333	45.994
	FXS Mosaics vs CSL	0.212	-3.958	0.000
	CS vs CSF	0.128	-0.0200	1.567
	CS vs CSL	0.029	-3.241	-0.357
	CSF vs CSL	0.001	1.210	3.958
Swing (%)	FXS Full Mutation vs FXS Mosaics	0.920	-2.051	4.074
	FXS Full Mutation vs CS	0.000	4.193	8.223
	FXS Full Mutation vs CSF	0.000	-8.851	-4.808
	FXS Full Mutation vs CSL	0.015	-6.548	-1.786
	FXS Mosaics vs CS	0.000	5.735	9.150
	FXS Mosaics vs CSF	0.000	-9.489	-6.667

	FXS Mosaics vs CSL	0.022	3.333	7.083
	CS vs CSF	0.246	-1.709	0.372
	CS vs CSL	0.025	0.754	3.819
	CSF vs CSL	0.002	-4.242	-1.494
Cadence (step/min)	FXS Full Mutation vs FXS Mosaics	0.213	-12.069	1.935
	FXS Full Mutation vs CS	0.000	5.333	15.915
	FXS Full Mutation vs CSF	0.001	-12.632	-2.564
	FXS Full Mutation vs CSL	0.660	-8.882	4.286
	FXS Mosaics vs CS	0.003	2.217	8.602
	FXS Mosaics vs CSF	0.147	-7.162	2.069
	FXS Mosaics vs CSL	0.117	-5.455	0.000
	CS vs CSF	0.056	0.000	5.819
	CS vs CSL	0.000	6.221	10.417
	CSF vs CSL	0.001	-9.231	-1.935
