# Supplementary material for: Feasibility and Reliability Assessment of Video-Based Motion Analysis and Surface Electromyography in Children with Fragile X during Gait
Source: Sensors (Basel). 2021 Jul 12;21(14):4746. doi: 10.3390/s21144746 (PMC8309640; doi:10.3390/s21144746)

Figure A3a: Envelope of muscle activity during gait in FXS subjects and CS (median values and IQR), bilaterally for the following muscles: Tibialis Anterior, Rectus Femoris, Gastrocnemius Lateralis, Biceps Femoris. Horizontal bars represent intervals of gait cycle with significant differences (Kruskal-Wallis test with post-hoc Wilcoxon rank sum test with Bonferroni correction). FXS Full mutation (red), FXS Mosaics (green) and CS (grey). Colors refer to the online version of the figure.

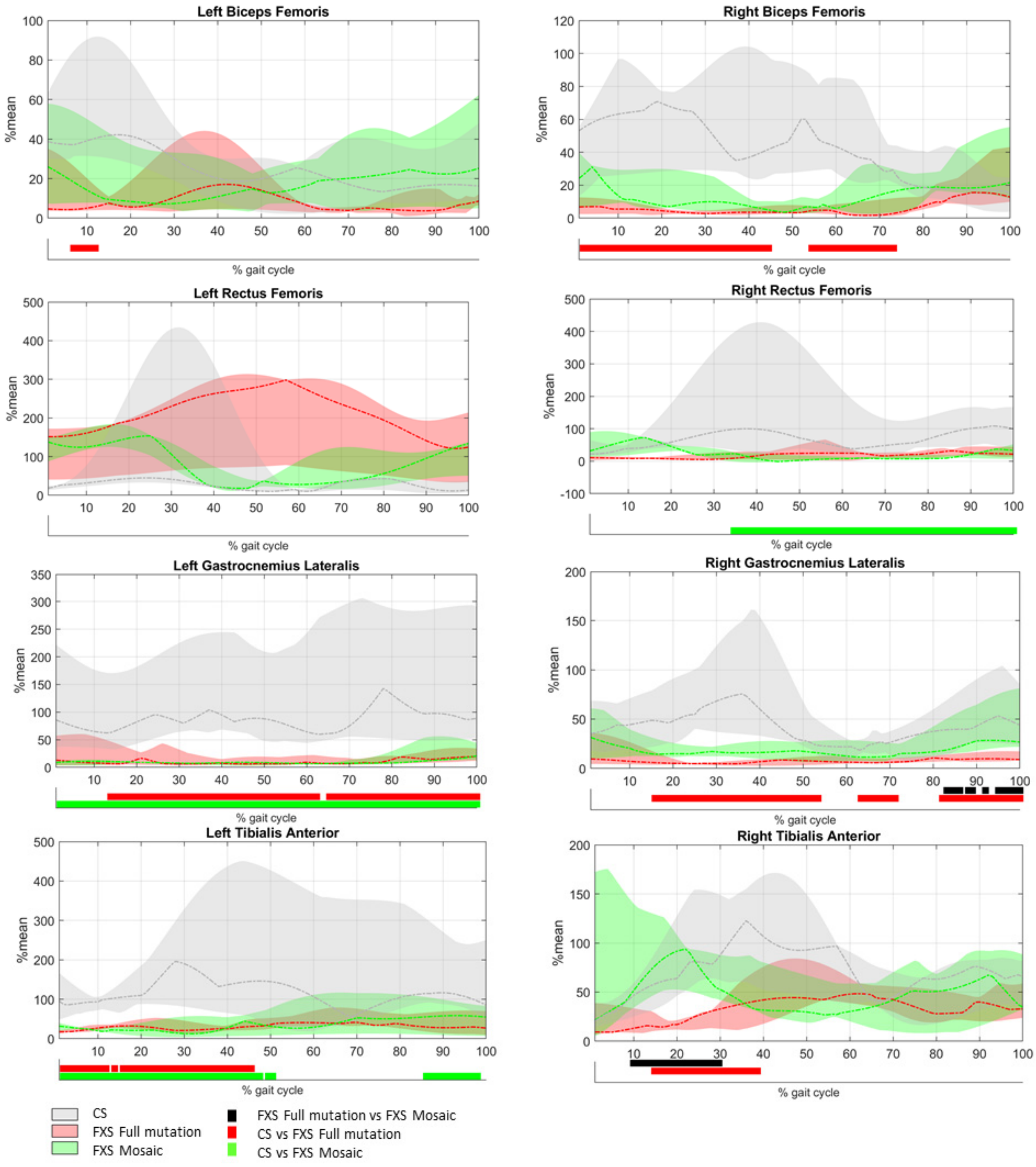

Figure A3b: Envelope of muscle activity during gait in FXS subjects and CS (median values and IQR), bilaterally for the following muscles: Tibialis Anterior, Rectus Femoris, Gastrocnemius Lateralis,

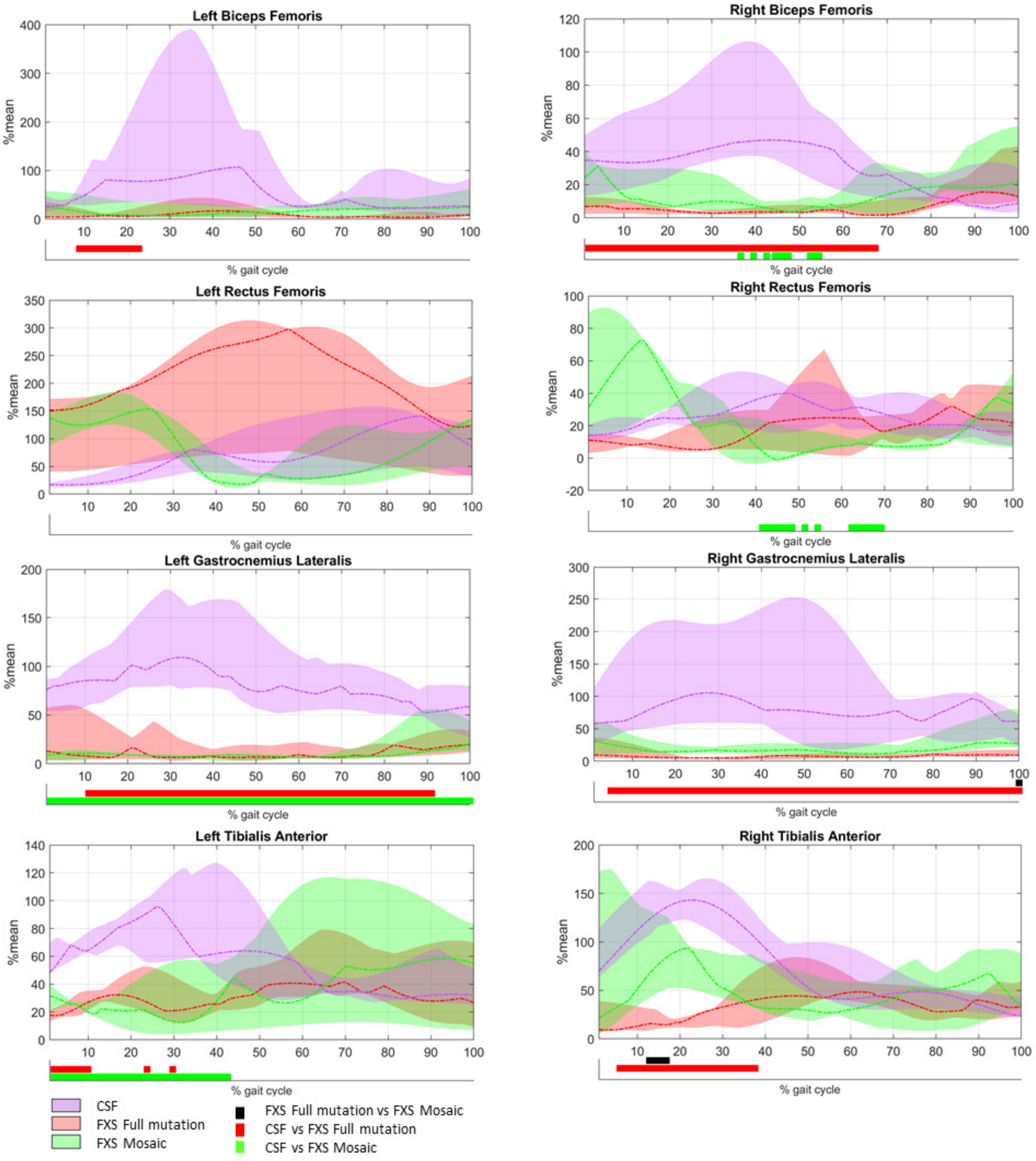

Biceps Femoris. Horizontal bars represent intervals of gait cycle with significant differences (Kruskal-Wallis test with post-hoc Wilcoxon rank sum test with Bonferroni correction). FXS Full mutation (red), FXS Mosaics (green) and CSF (purple). Colors refer to the online version of the figure.

Figure A3c: Envelope of muscle activity during gait in FXS subjects and CS (median values and IQR), bilaterally for the following muscles: Tibialis Anterior, Rectus Femoris, Gastrocnemius Lateralis, Biceps Femoris. Horizontal bars represent intervals of gait cycle with significant differences (Kruskal-

Wallis test with post-hoc Wilcoxon rank sum test with Bonferroni correction). FXS Full mutation (red), FXS Mosaics (green) and CSL (yellow). Colors refer to the online version of the figure

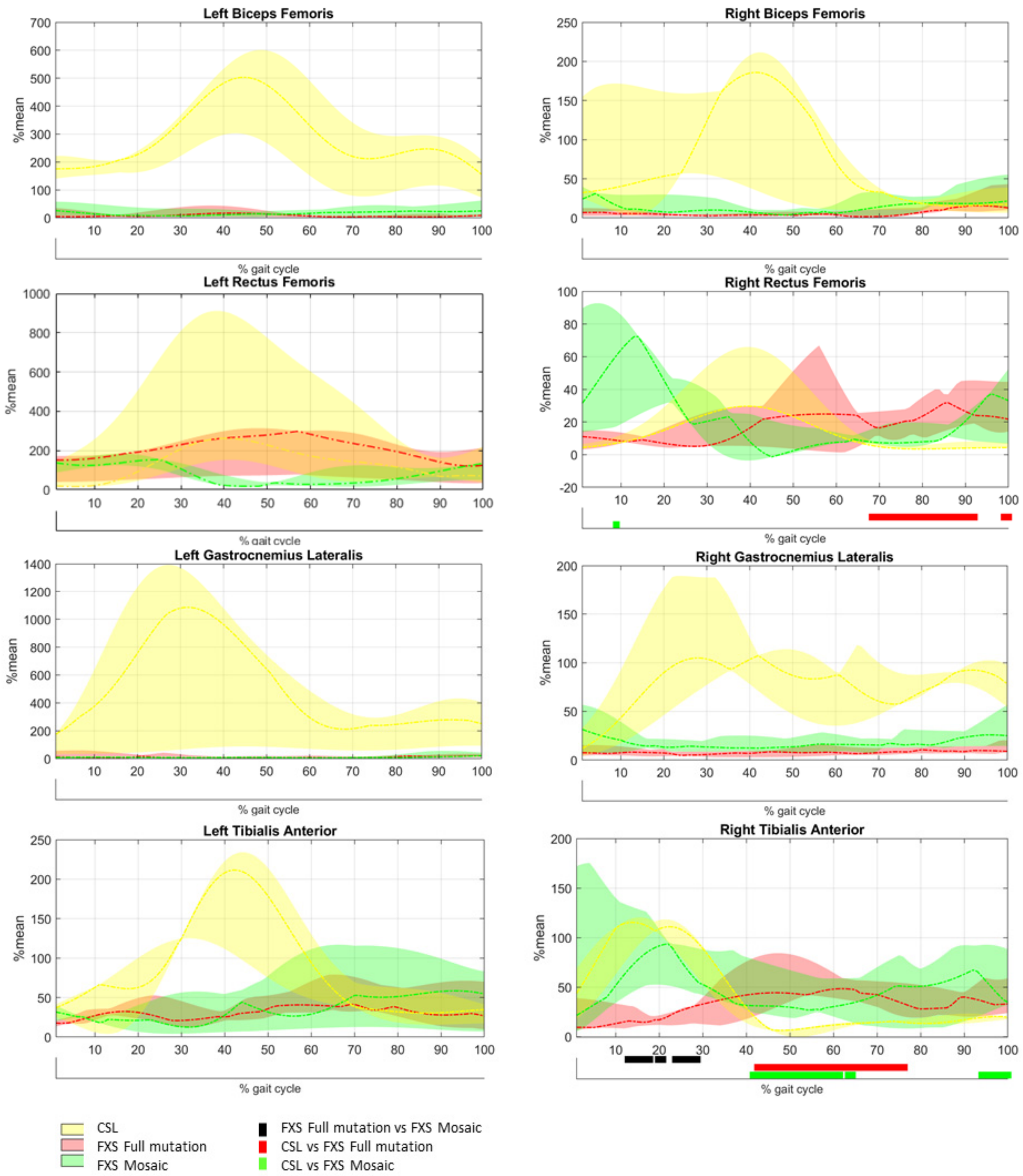

Supplement: Supplementary file 1 [file sensors-21-04746-s001.zip › Figure A3.pdf]
