# Supplementary material for: Feasibility and Reliability Assessment of Video-Based Motion Analysis and Surface Electromyography in Children with Fragile X during Gait
Source: Sensors (Basel). 2021 Jul 12;21(14):4746. doi: 10.3390/s21144746 (PMC8309640; doi:10.3390/s21144746)
Supplement: Supplementary file 1 [file sensors-21-04746-s001.zip › Table A1a.pdf]

Table A1a. Gait velocity in the preliminary set of CS [19], mean and standard deviation in the analysed subjects.

CS Subjects	Trial	Velocity [m/s]
CS1	DX1	1,08
	DX2	1,01
	DX3	1,03
	SX1	1,13
	SX2	1,06
	SX3	1,07
CS2	DX1	0,95
	DX2	1,00
	DX3	1,01
	SX1	0,98
	SX2	0,98
	SX3	1,06
CS3	DX1	0,88
	DX2	0,89
	DX3	0,96
	SX1	1,00
	SX2	0,86
	SX3	1,03
CS4	DX1	1,23
	DX2	1,20
	DX3	1,18
	SX1	1,19
	SX2	1,14
	SX3	1,05
CS5	DX1	1,24
	DX2	1,21
	DX3	1,27
	SX1	1,31
	SX2	1,32
	SX3	1,25
CS6	DX1	1,11
	SX1	1,16
CS7	DX1	1,14
	DX2	1,08
	DX3	1,14
	SX1	1,23
	SX2	1,27
	SX3	0,95
CS8	DX1	1,25
	DX2	0,99
	DX3	1,12
	SX1	1,17
	SX2	0,93
	SX3	1,07
CS9	DX1	1,20
	DX2	1,11

	DX3	1,14
	SX1	1,22
	SX2	1,10
	SX3	1,10
CS10	DX1	1,05
	SX1	1,15
**MEAN**	**1,10**	
**ST. DEV.**	**0,12**	

Table A1b. Gait velocity in the preliminary set of FXS [19], mean and standard deviation in the analysed subjects.

FXS Subjects	Trial	Velocity [m/s]
FXS1	DX1	0,59
	SX1	0,81
FXS2	DX1	1,03
	DX2	0,99
	SX1	0,97
	SX2	1,06
FXS3	DX1	0,72
	SX1	0,63
FXS4	DX1	0,56
	DX2	0,50
	SX1	0,69
	SX2	1,14
**MEAN**	**0,81**	
**ST. DEV.**	**0,22**	
