# Supplementary figures and images for: Feasibility and Reliability Assessment of Video-Based Motion Analysis and Surface Electromyography in Children with Fragile X during Gait
Source: Sensors (Basel). 2021 Jul 12;21(14):4746. doi: 10.3390/s21144746 (PMC8309640; doi:10.3390/s21144746)

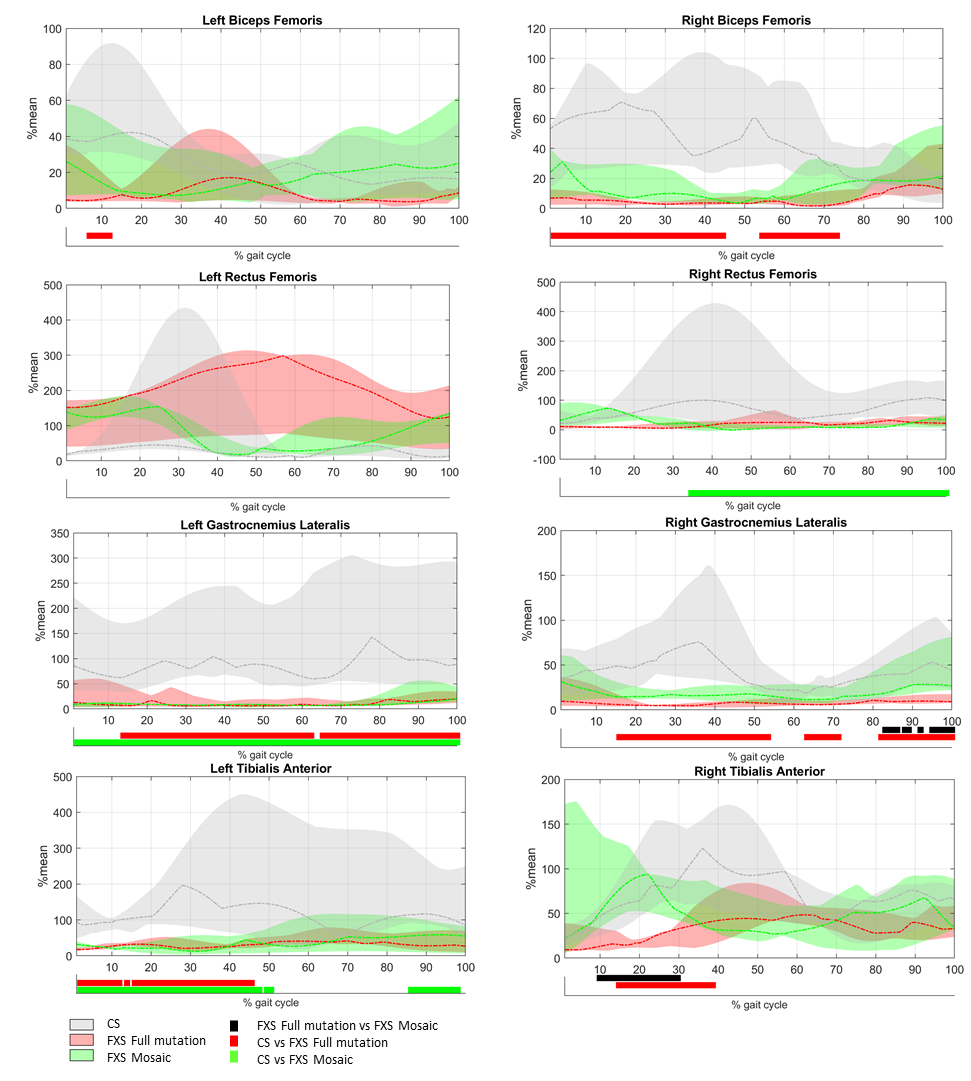

Supplement: Supplementary file 1 [file sensors-21-04746-s001.zip › Figure A2a.PNG]

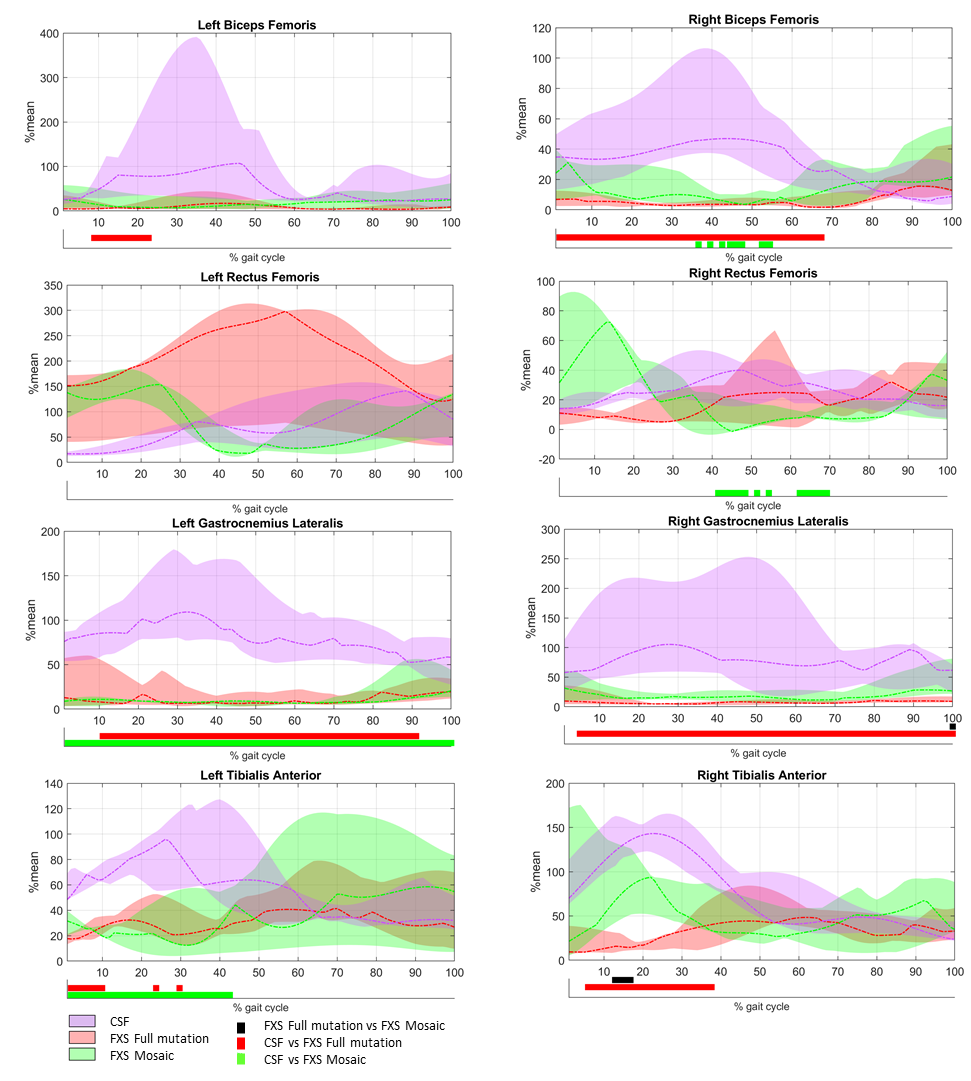

Supplement: Supplementary file 1 [file sensors-21-04746-s001.zip › Figure A2b.PNG]

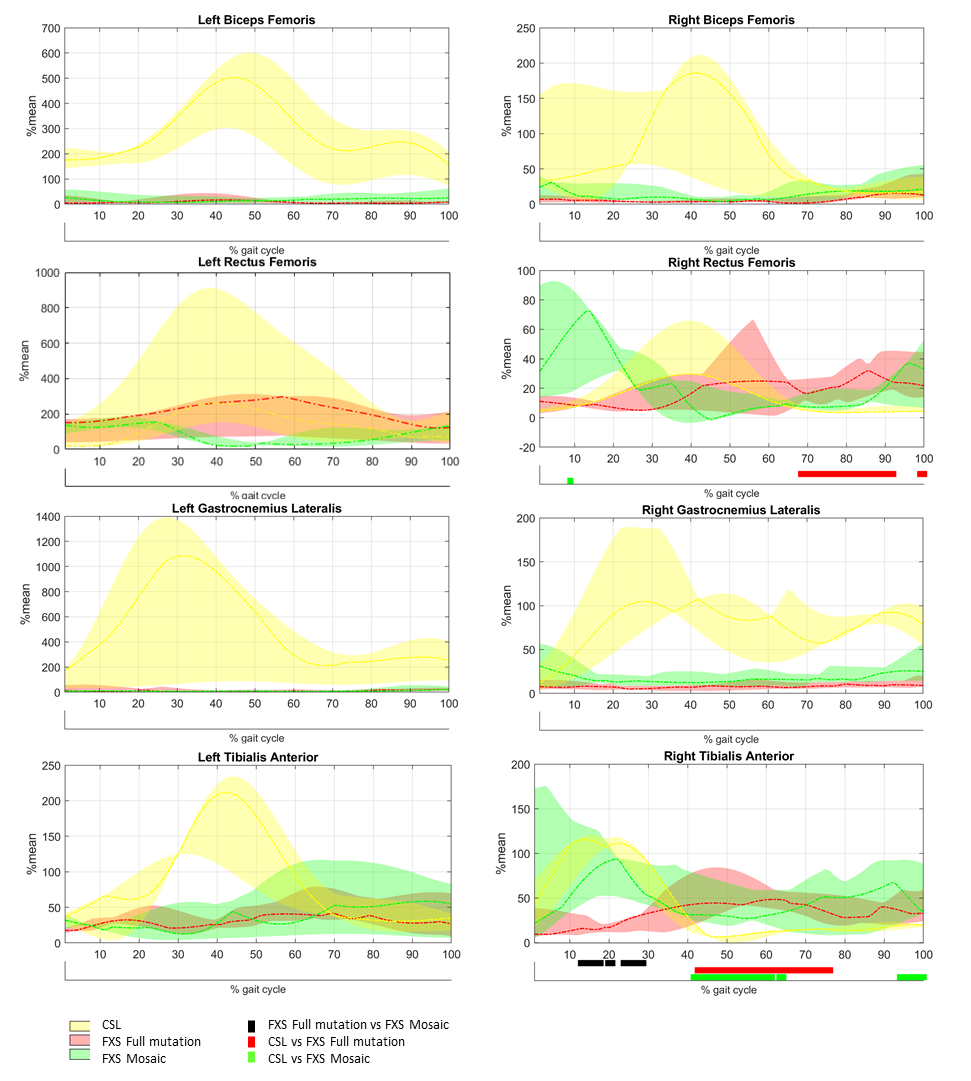

Supplement: Supplementary file 1 [file sensors-21-04746-s001.zip › Figure A2c.PNG]

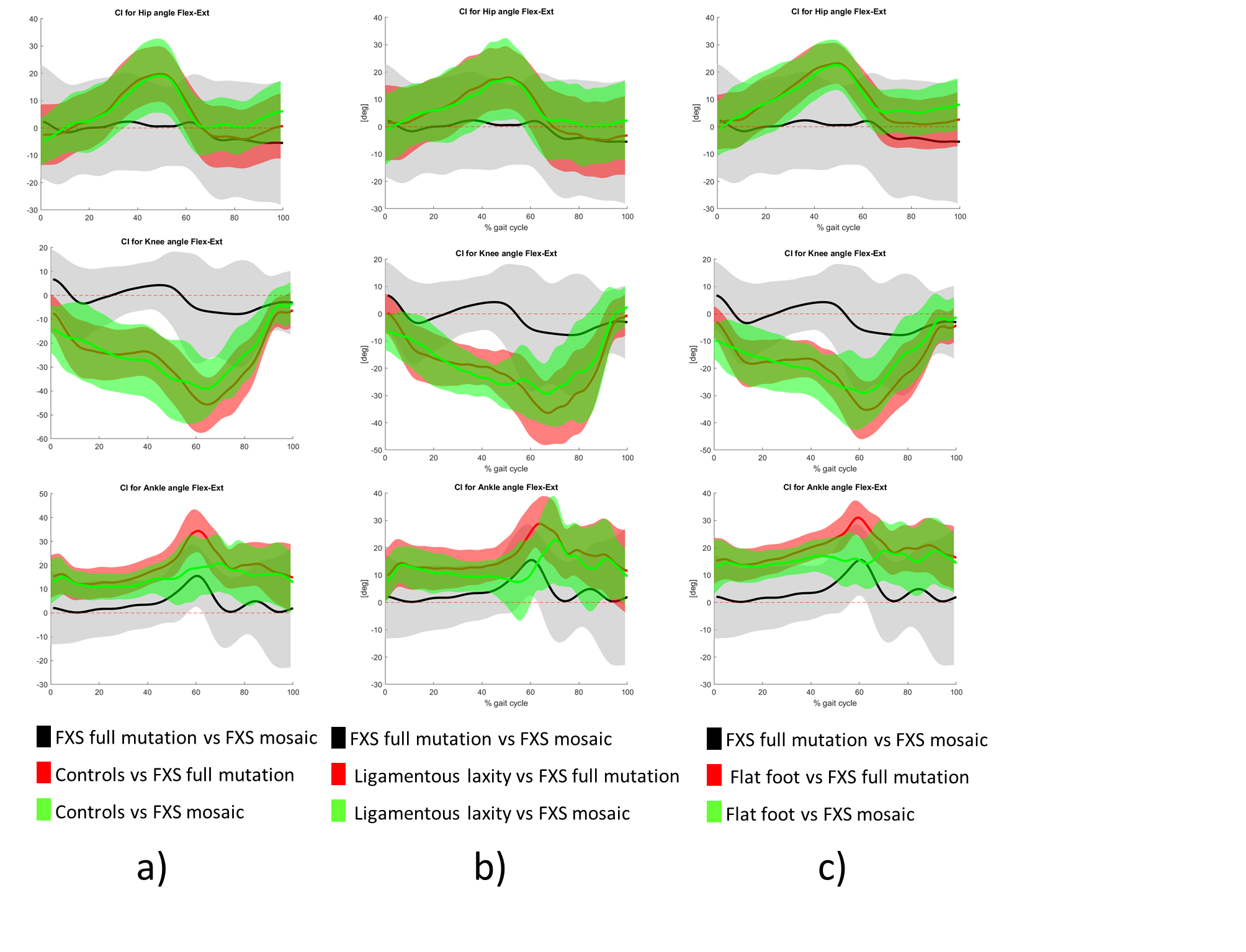

Supplement: Supplementary file 1 [file sensors-21-04746-s001.zip › FigureA3a.png]
